# Supplementary material for: Chemical Constituents with Inhibitory Activity of NO Production from a Wild Edible Mushroom, Russula vinosa Lindbl, May Be Its Nutritional Ingredients
Source: Molecules. 2019 Apr 3;24(7):1305. doi: 10.3390/molecules24071305 (PMC6479787; doi:10.3390/molecules24071305)
Supplement: Supplementary file 1 [file molecules-24-01305-s001.zip › molecules-474663-SI.pdf]

Chemical constituents with inhibitory activity of NO production from a wild edible mushroom, *Russula vinosa* Lindbl, may be its nutritional ingredients

Guodong Zhang [1], Huawei Geng [2], Chunxia Zhao [1], Fangyi Li [1], Zhen-Fa Li [1], Boshu Lun [1], Chunhua Wang [1,3,*] Heshui Yu [1], Songtao Bie [1] and Zheng Li [1,3,*]

[1] College of Pharmaceutical Engineering of Traditional Chinese Medicine, Tianjin University of Traditional Chinese Medicine, Tianjin 300193, China; pharmgd@126.com (G.Z.); zhaochunxia199411@163.com (C.Z.); li_fangyi@hotmail.com (F.L.); lizhenxiaofa@163.com (Z.-F.L.); lbsxcl@163.com (B.L.); hs_yu08@163.com (H.Y.); biesongtao@126.com (S.B.)

[2] Chongqing Municipal Public Security Bureau Evidence Identification Center, Chongqing 400707, China; huaweigeng0414@sina.com

[3] Tianjin Key Laboratory of Modern Chinese Medicine, Tianjin University of Traditional Chinese Medicine, Tianjin 300193, China

* Correspondence: pharmwch@126.com (C.W.); lizheng@tjutcm.edu.cn (Z.L.); Tel./Fax: +86-22-5979-1813 (C.W.)

Supplementary Materials

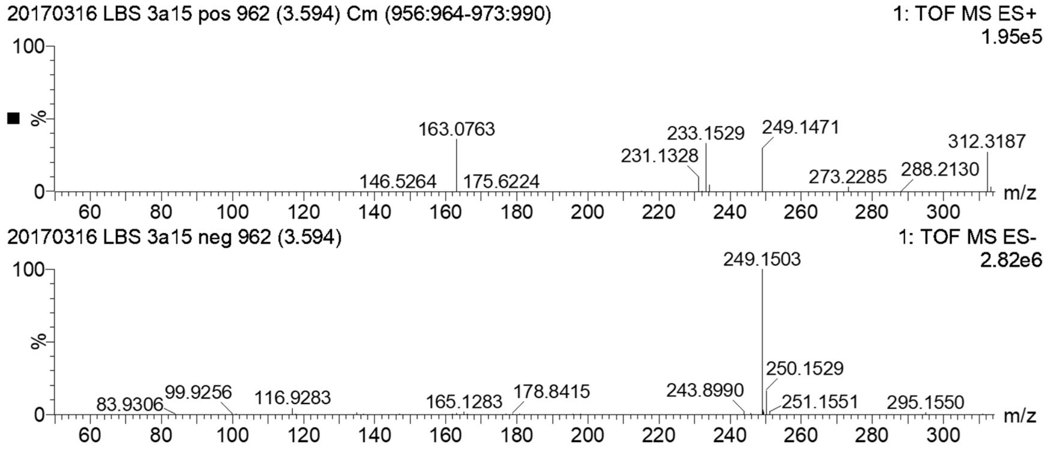

Figure S1. HR-ESI-MS spectrum of compound **1**

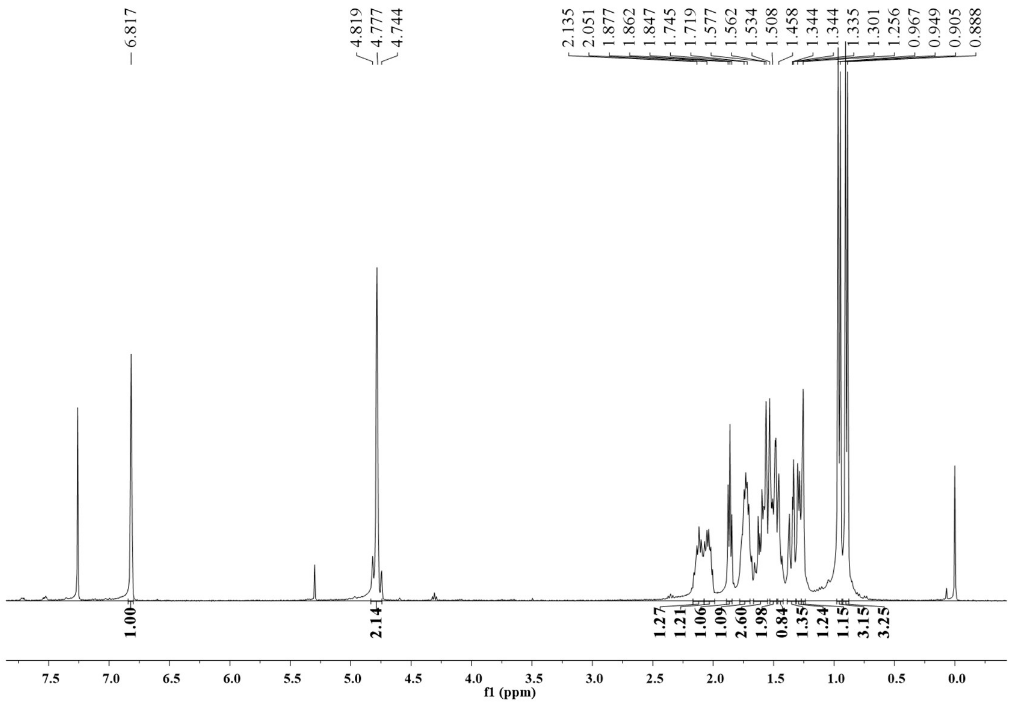

Figure S2. ^{1}H-NMR spectrum of compound **1**

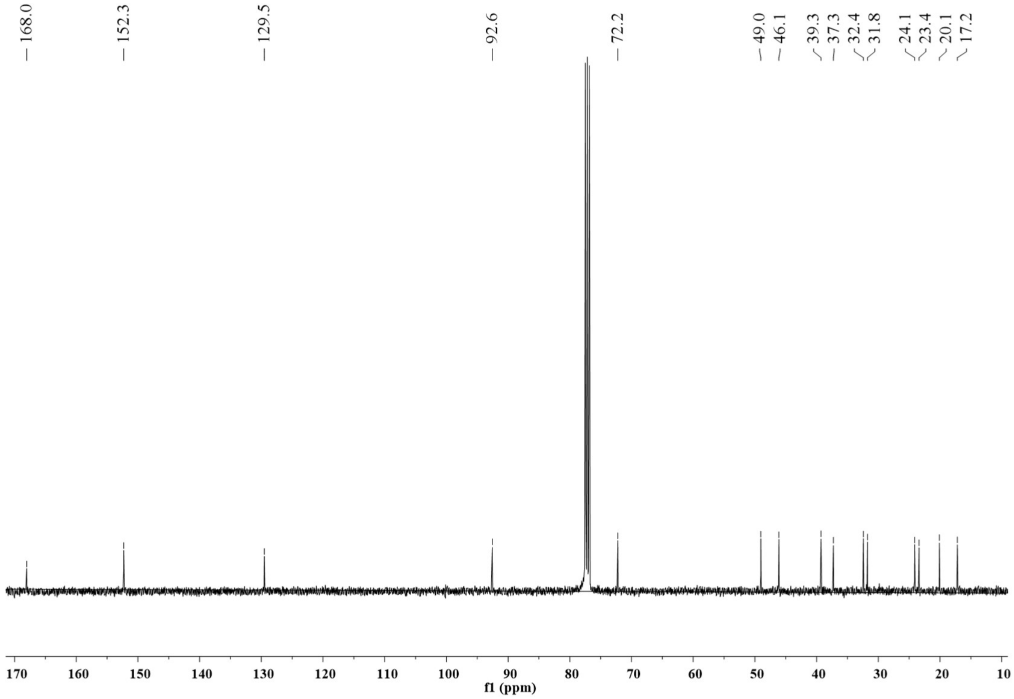

Figure S3. ^{13}C-NMR spectrum of compound **1**

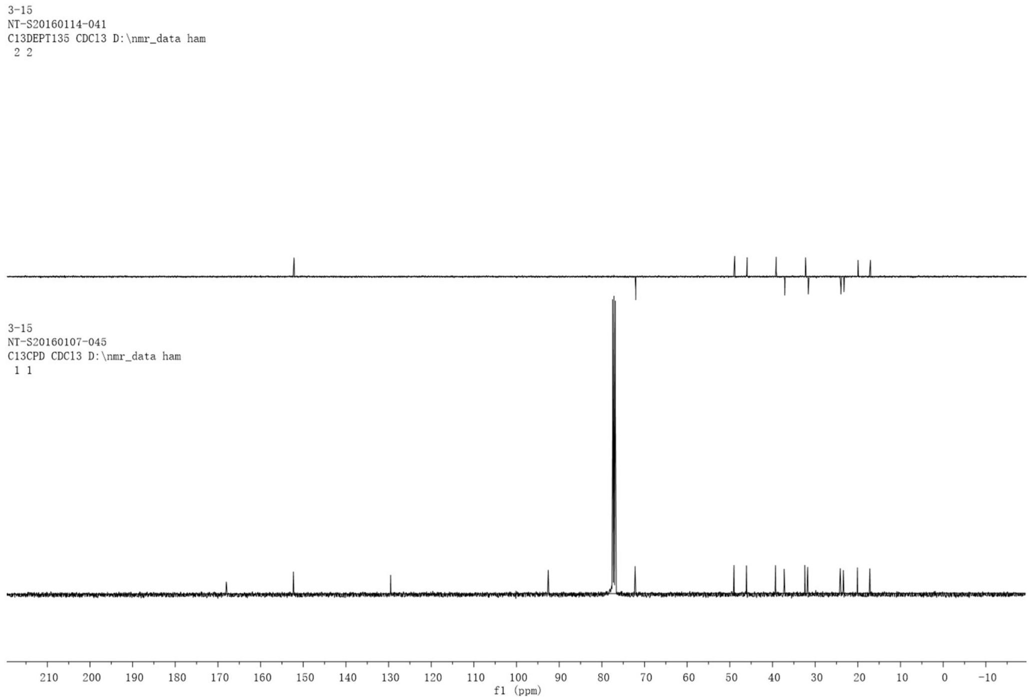

Figure S4. DEPT-135 spectrum of compound **1**

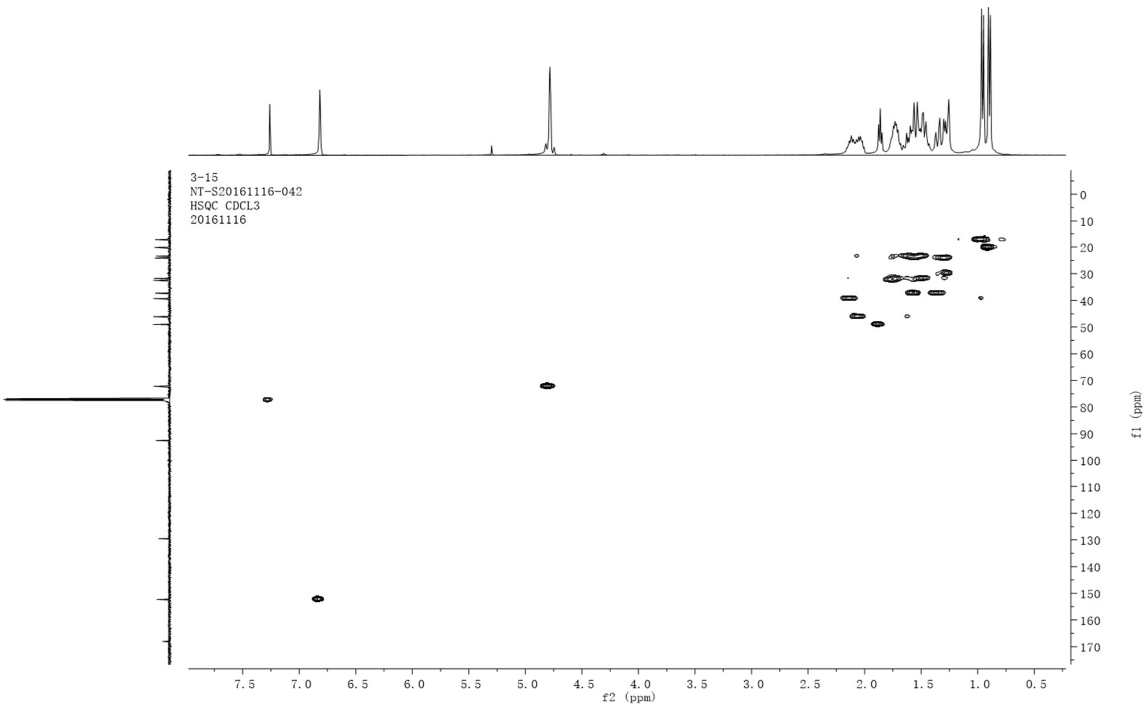

Figure S5. HSQC spectrum of compound **1**

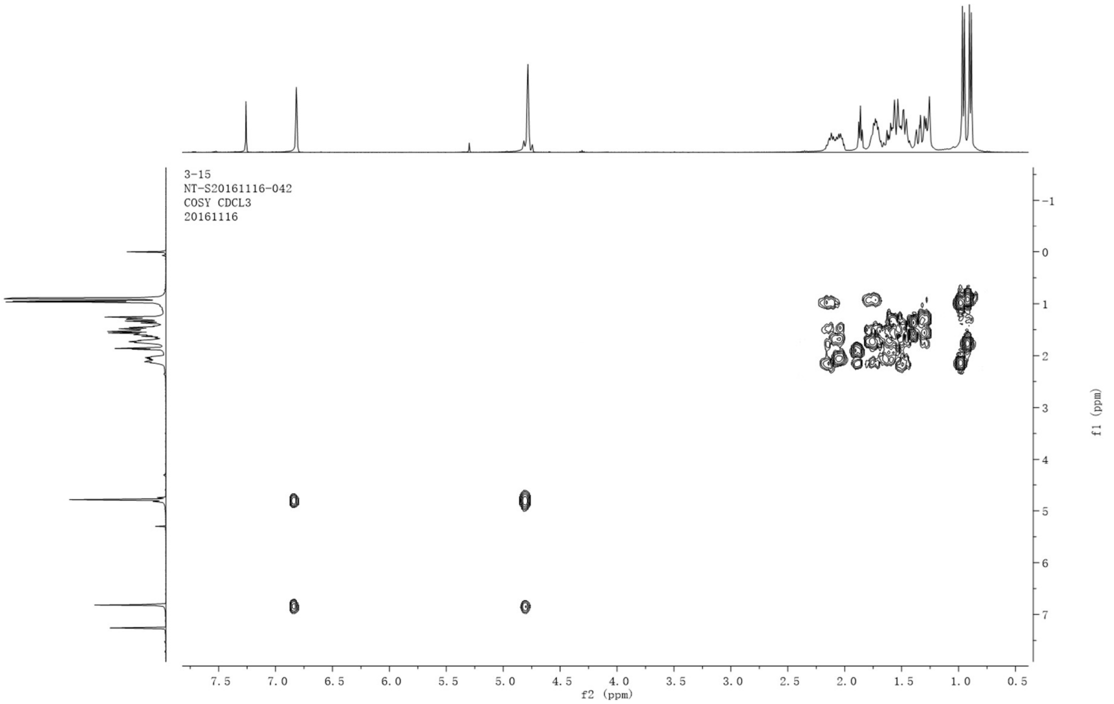

Figure S6. ^{1}H-^{1}H COSY spectrum of compound **1**

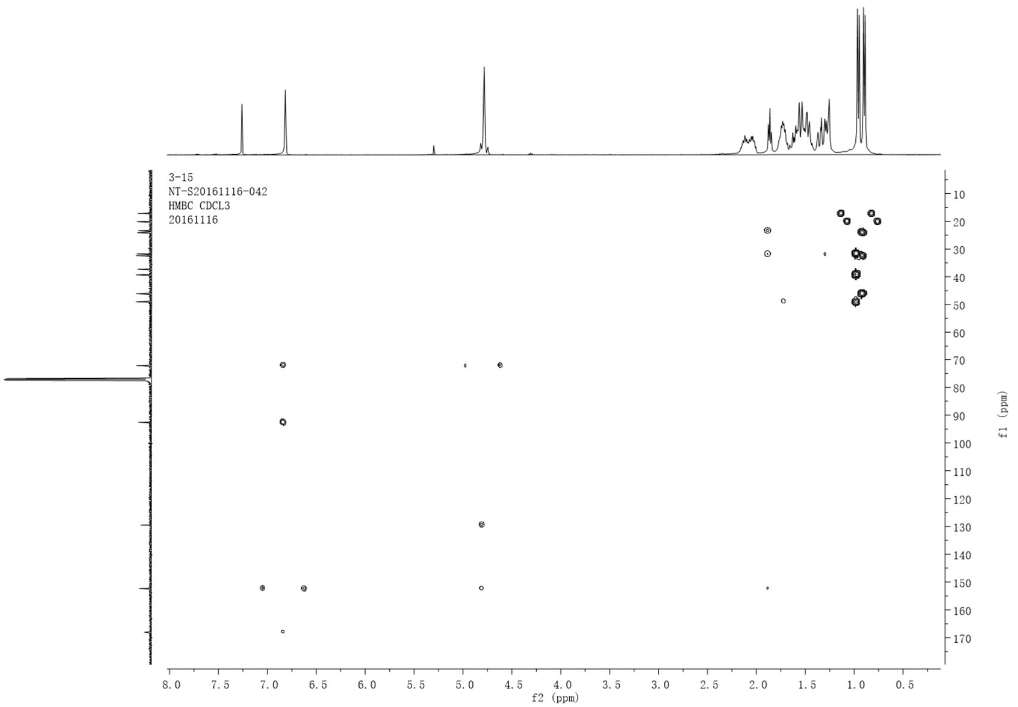

Figure S7. HMBC spectrum of compound **1**

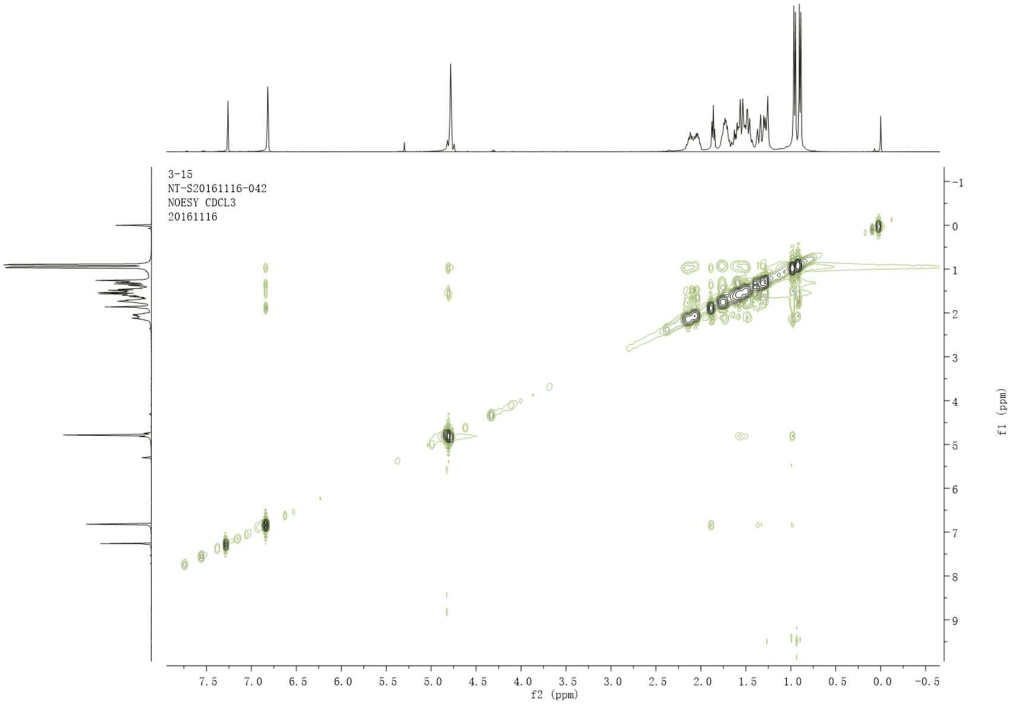

Figure S8. NOESY spectrum of compound **1**

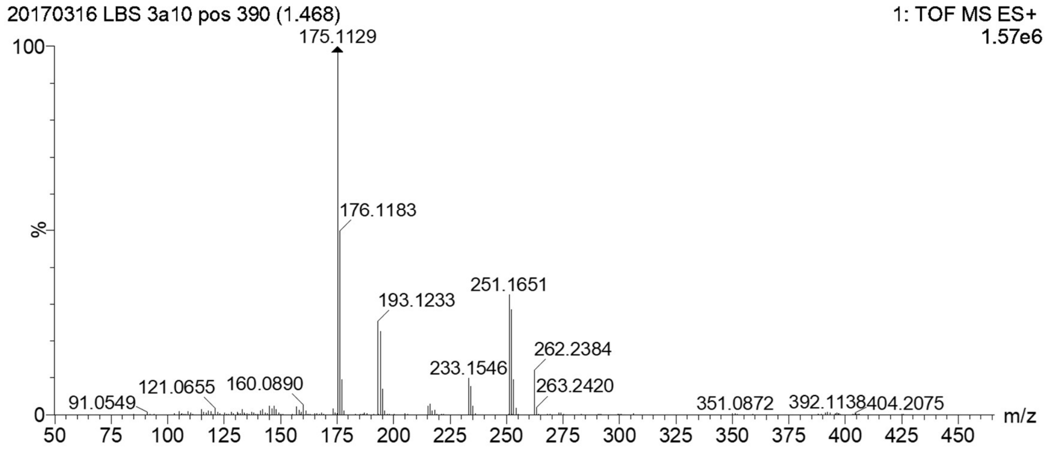

Figure S9. HR-ESI-MS spectrum of compound **2**

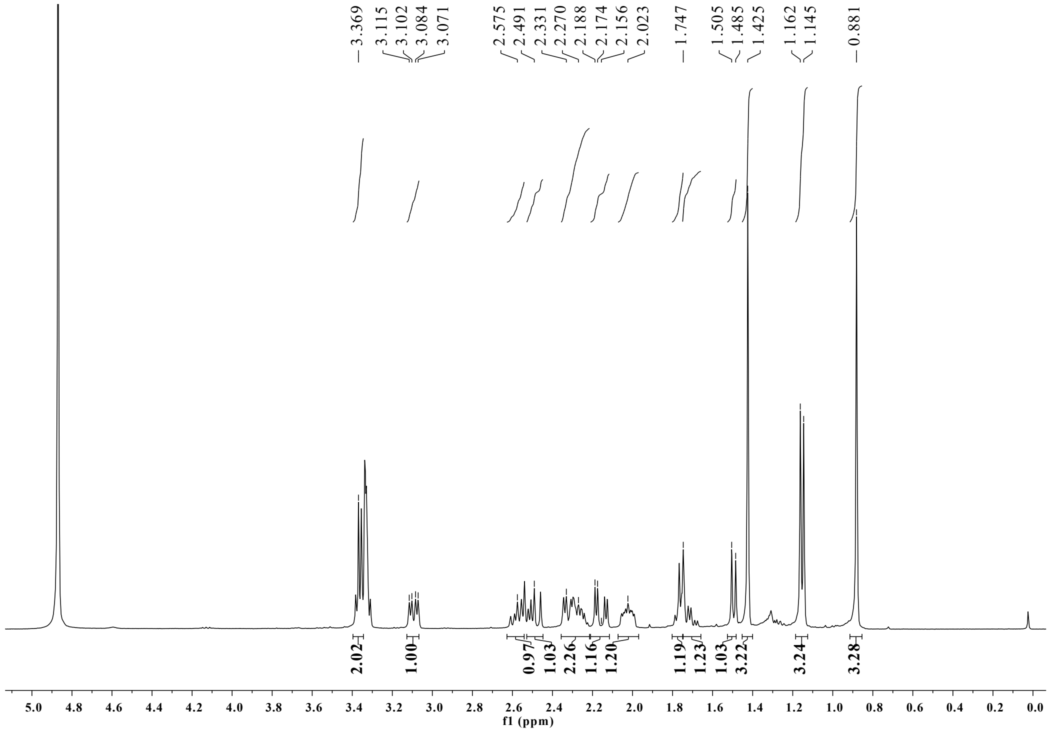

Figure S10. ^{1}H-NMR spectrum of compound **2**

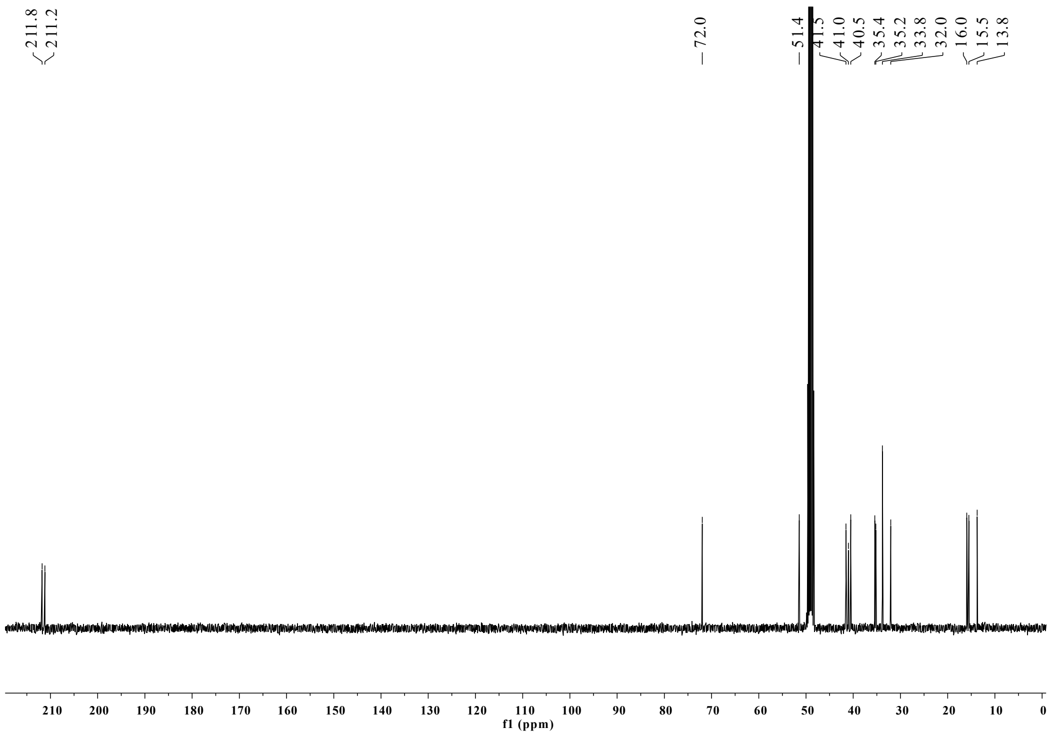

Figure S11. ^{13}C-NMR spectrum of compound **2**

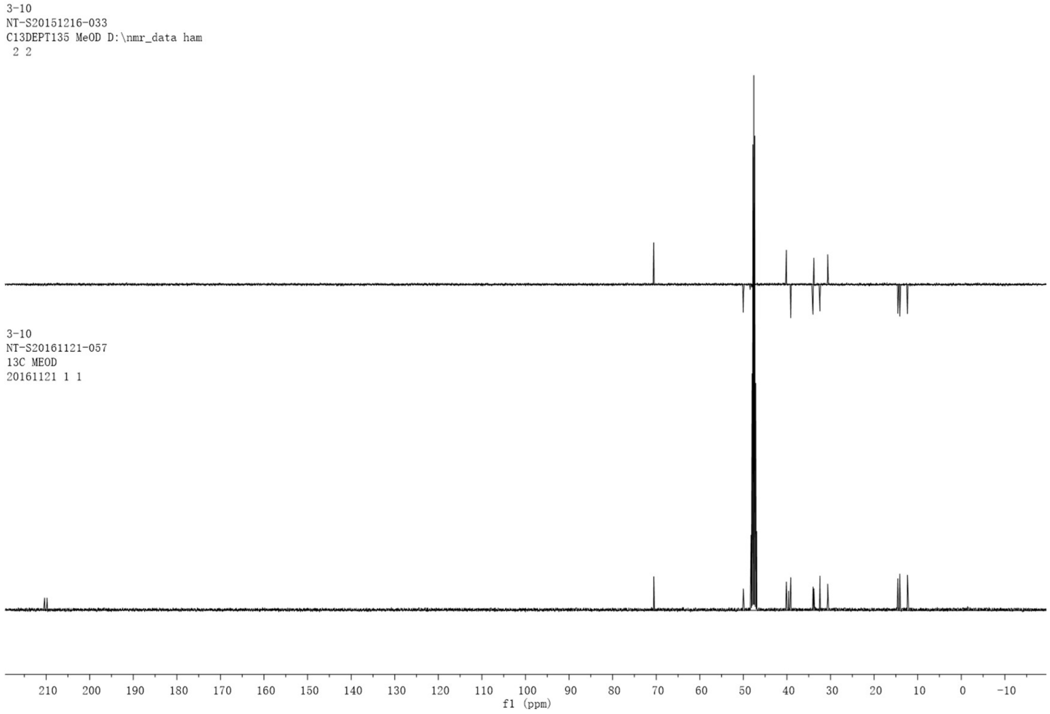

Figure S12. DEPT-135 spectrum of compound **2**

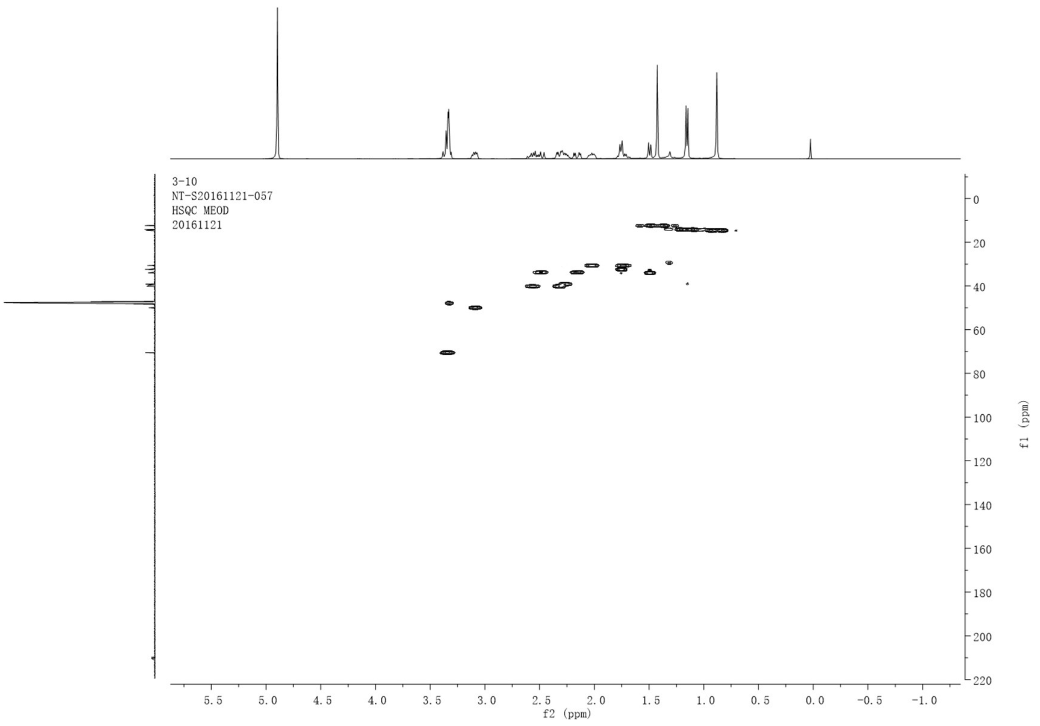

Figure S13. HSQC spectrum of compound **2**

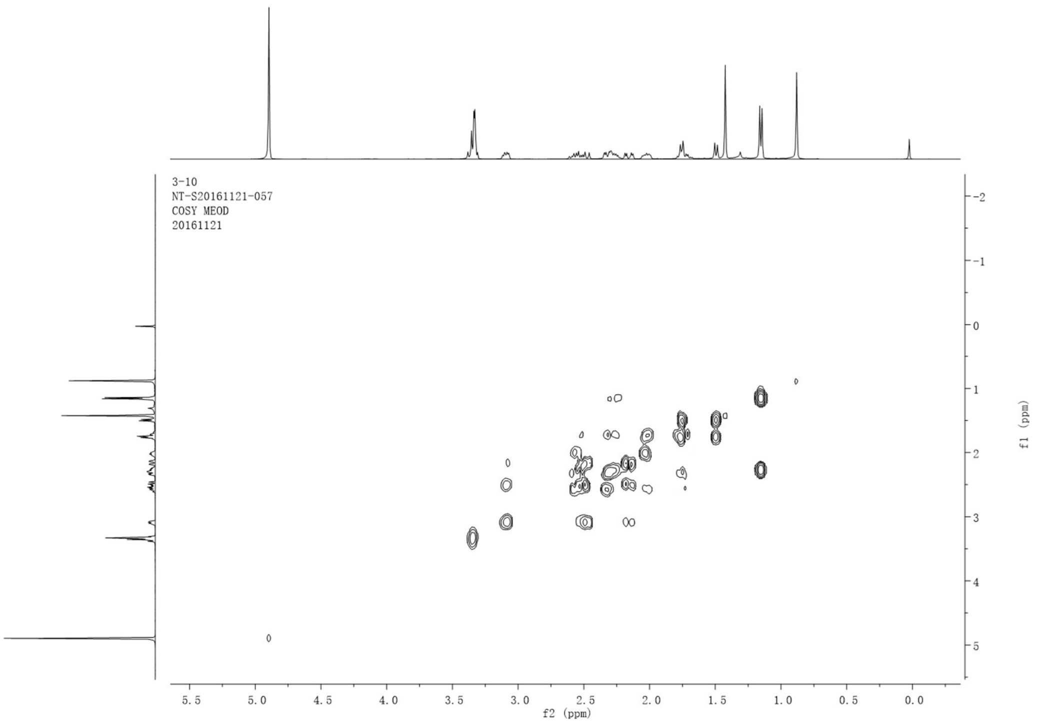

Figure S14. ^{1}H-^{1}H COSY spectrum of compound **2**

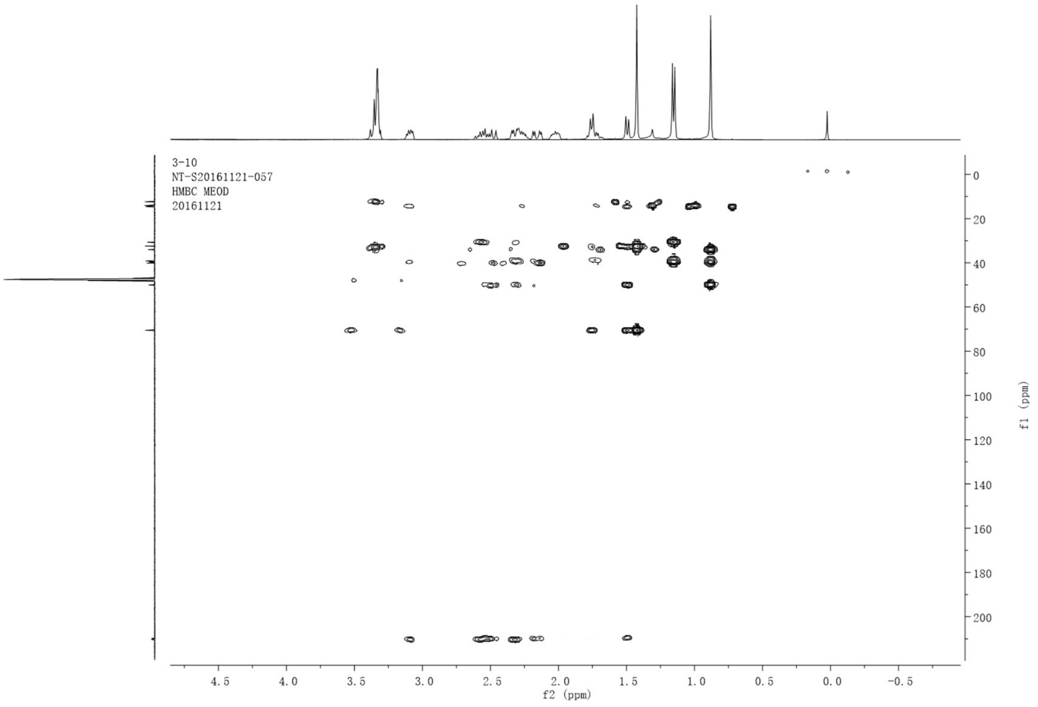

Figure S15. HMBCspectrum of compound **2**

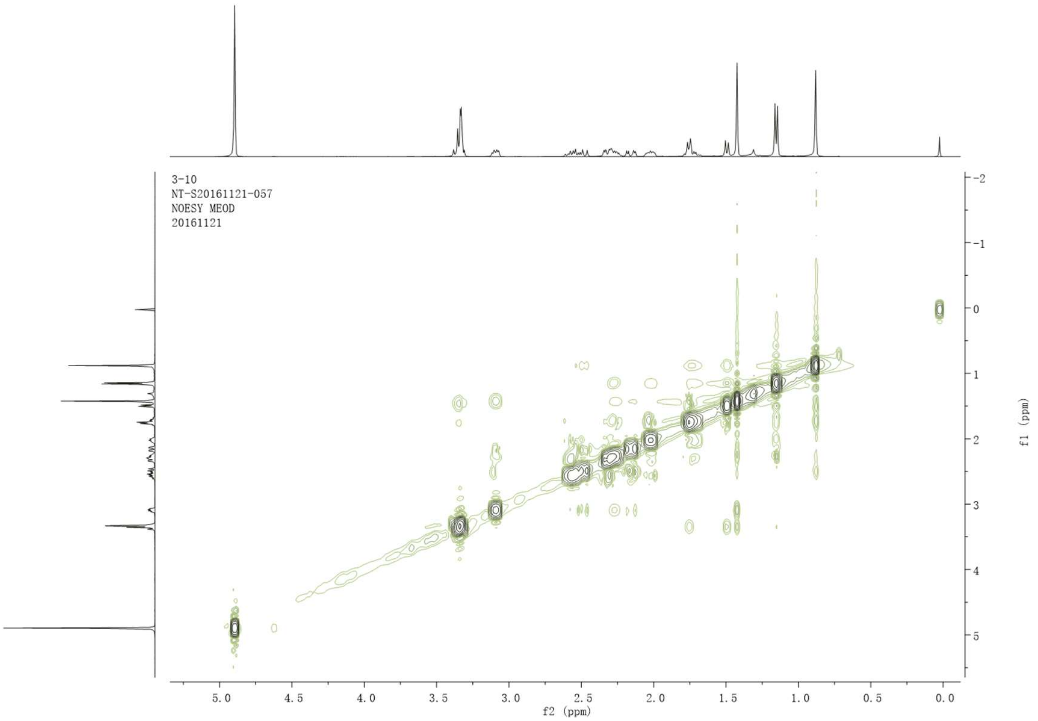

Figure S16. NOESY spectrum of compound **2**

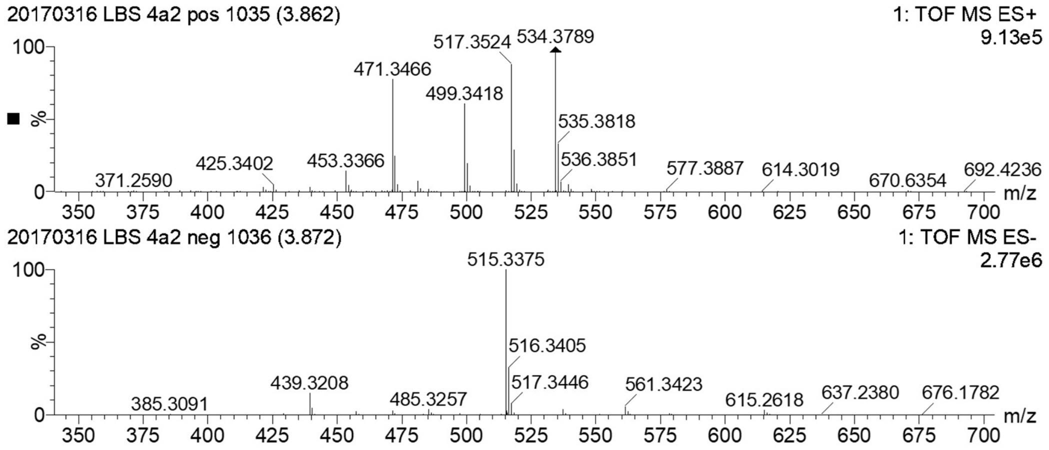

Figure S17. HR-ESI-MS spectrum of compound **3**

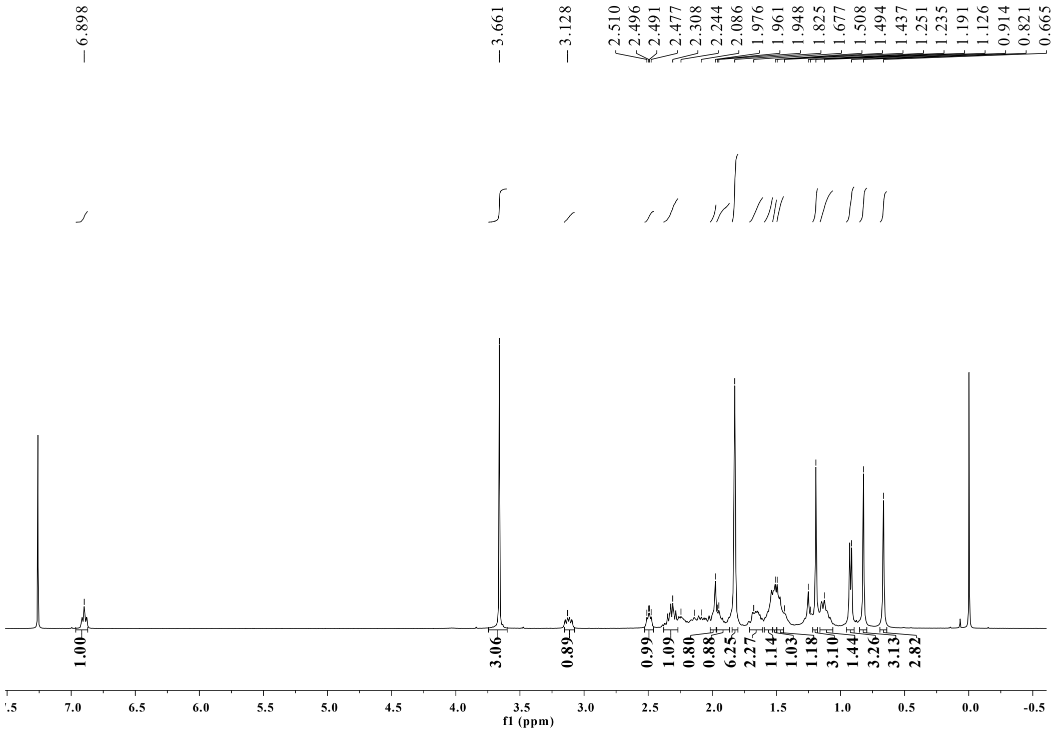

Figure S18. ^{1}H-NMR spectrum of compound **3**

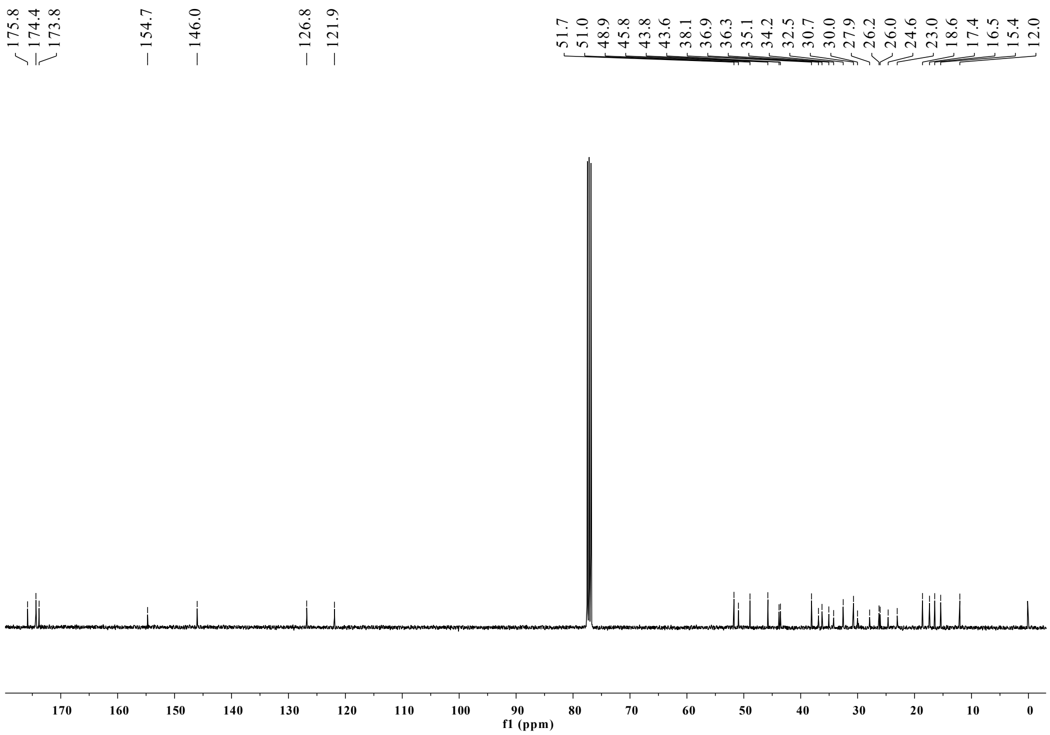

Figure S19. ^{13}C-NMR spectrum of compound **3**

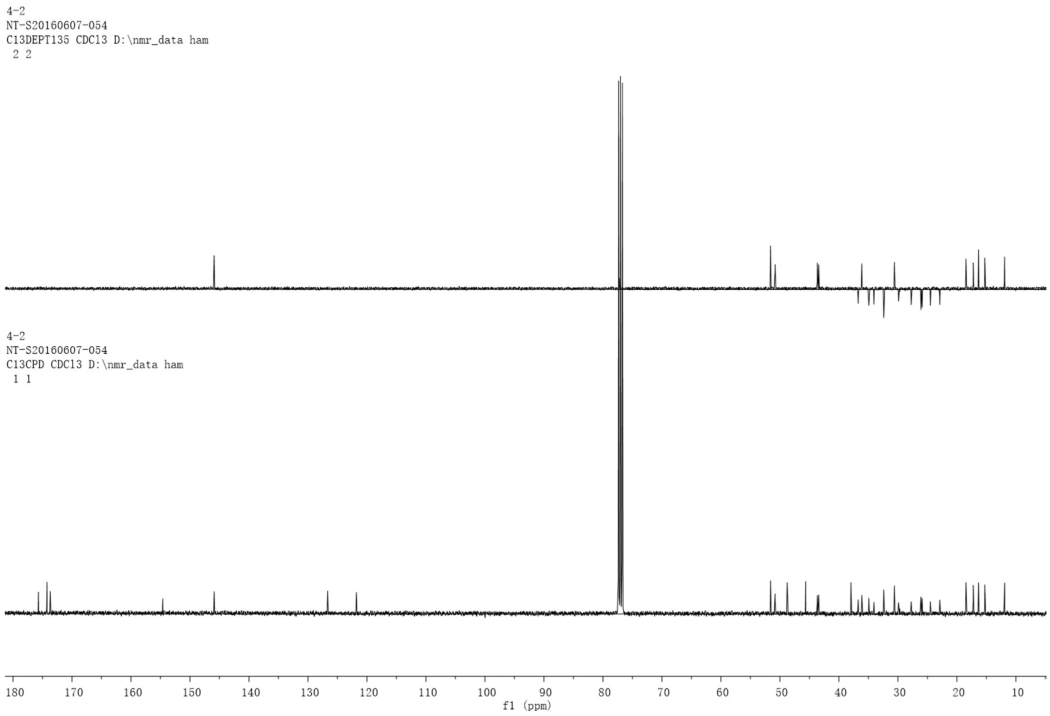

Figure S20. DEPT-135 spectrum of compound **3**

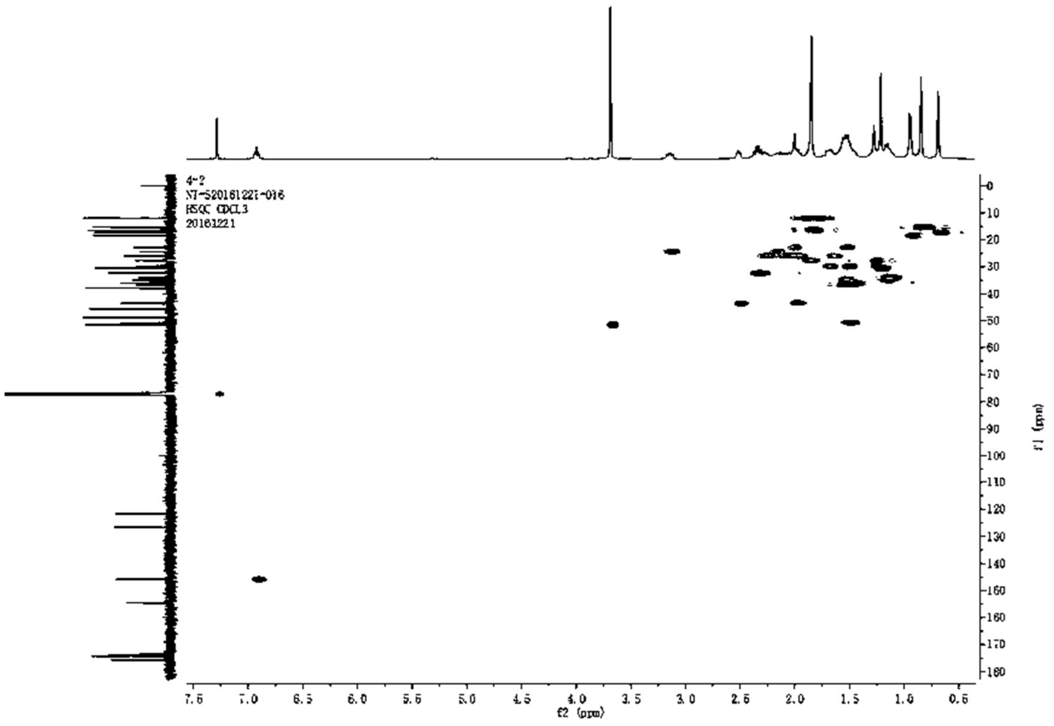

Figure S21. HSQC spectrum of compound **3**

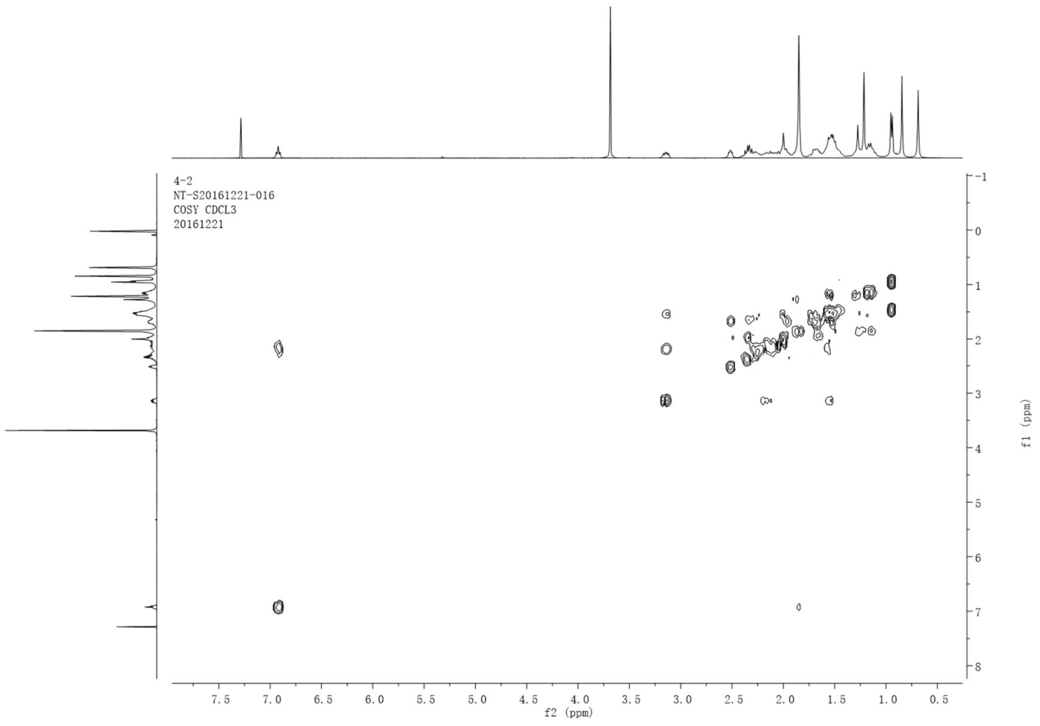

Figure S22. ^{1}H-^{1}H COSY spectrum of compound **3**

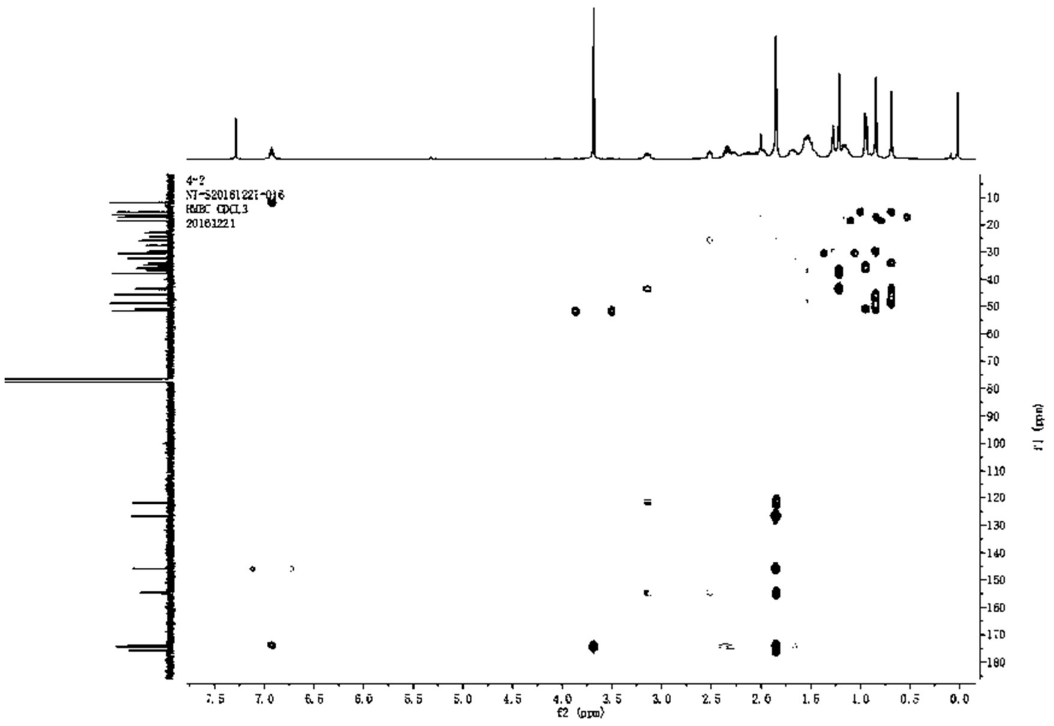

Figure S23. HMBC spectrum of compound **3**

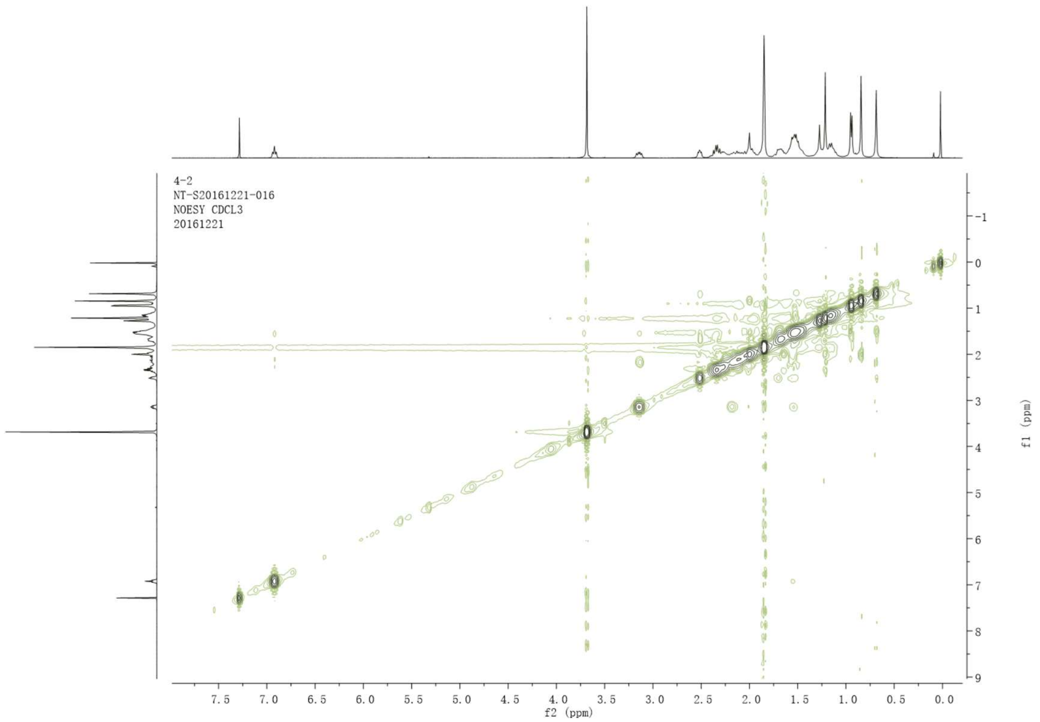

Figure S24. NOESY spectrum of compound **3**

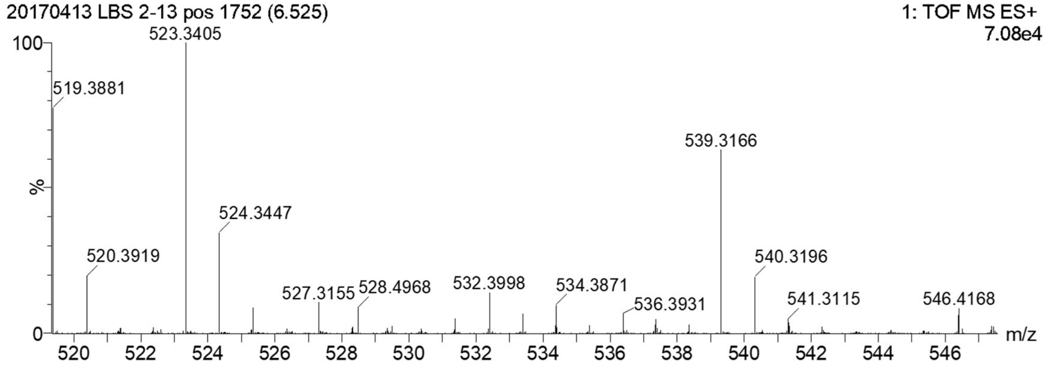

Figure S25. HR-ESI-MS spectrum of compound **4**

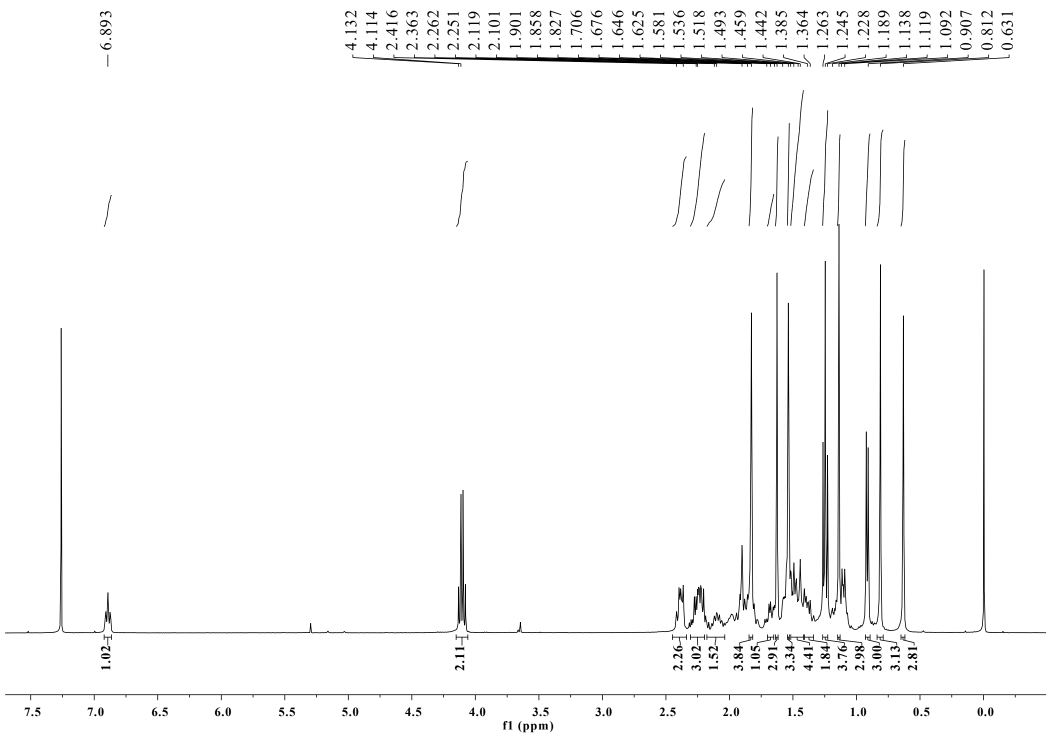

Figure S26. ^{1}H-NMR spectrum of compound **4**

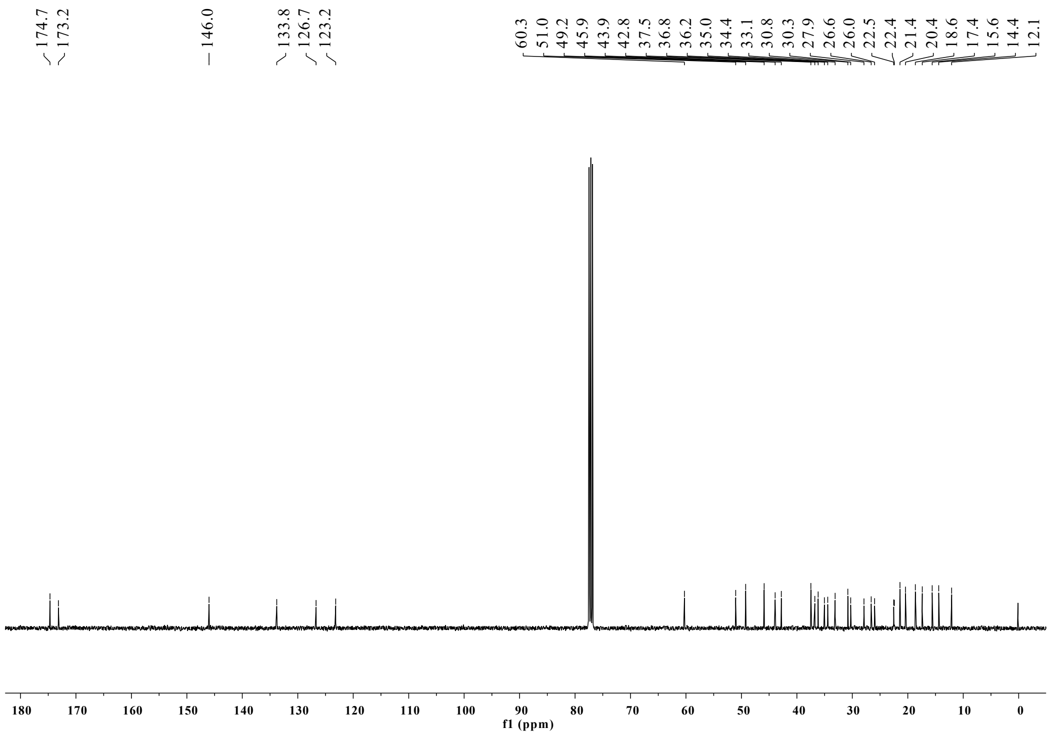

Figure S27. ¹³C-NMR spectrum of compound **4**

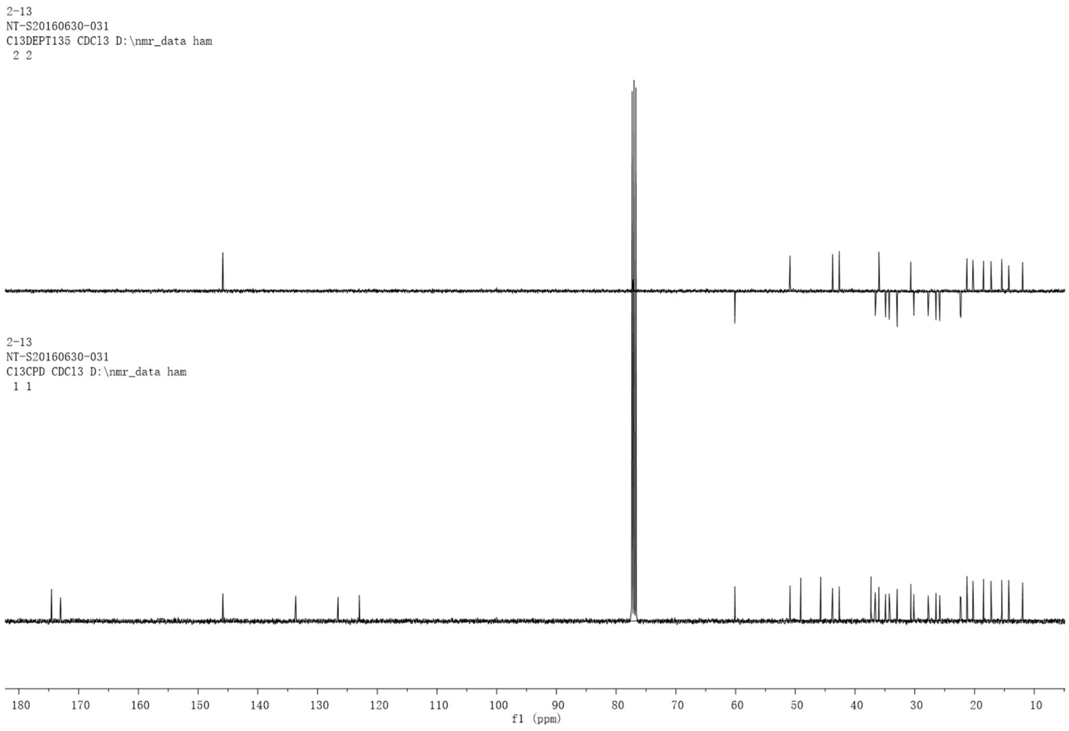

Figure S28. DEPT-135 spectrum of compound **4**

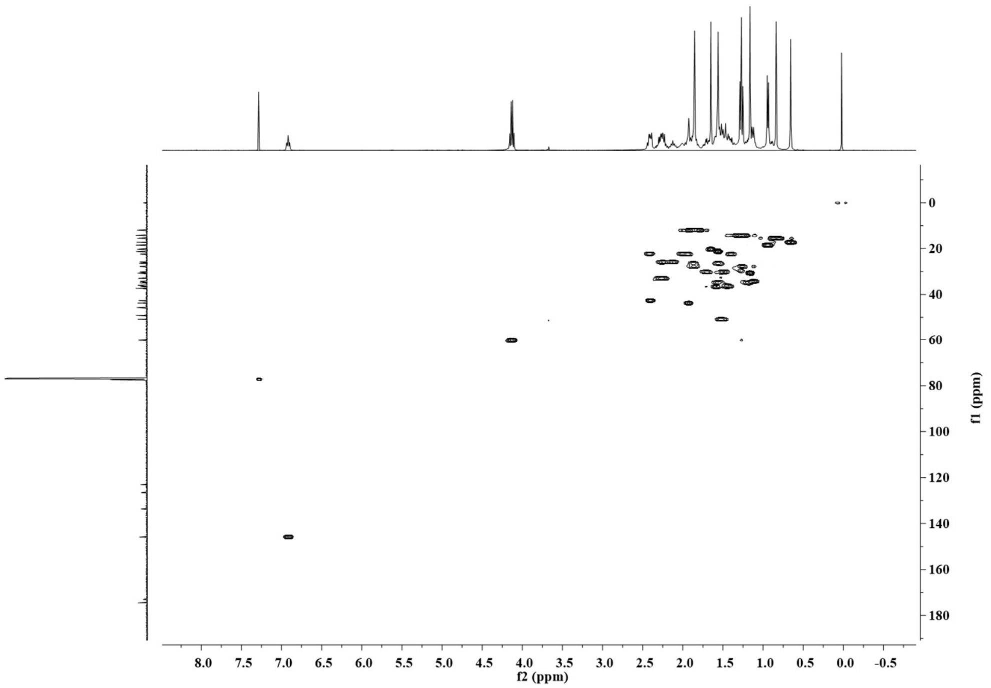

Figure S29. HSQC spectrum of compound **4**

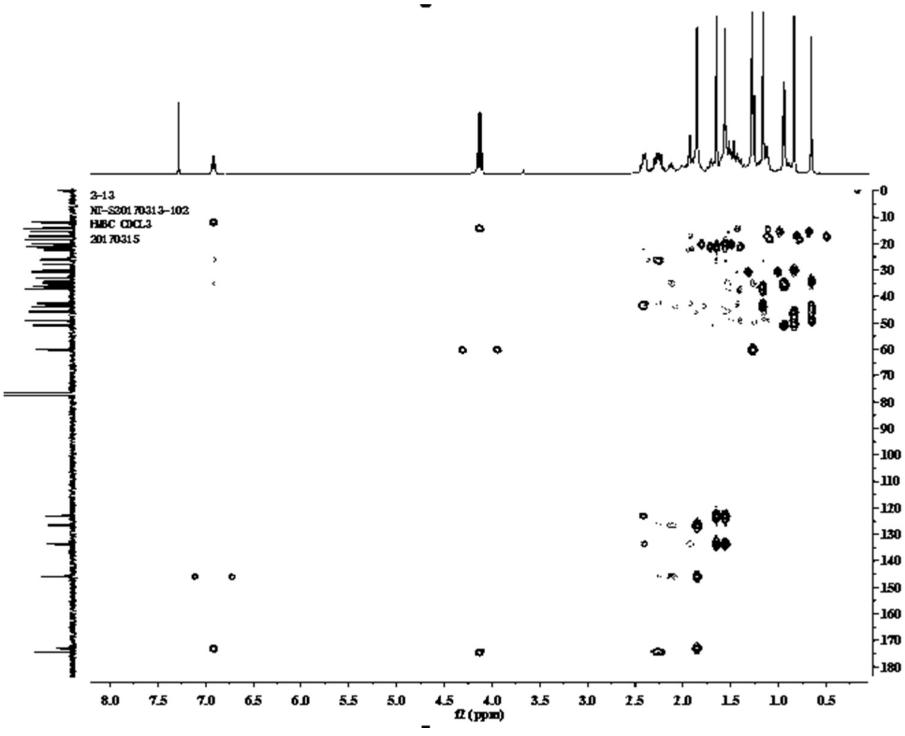

Figure S30. HMBC spectrum of compound **4**

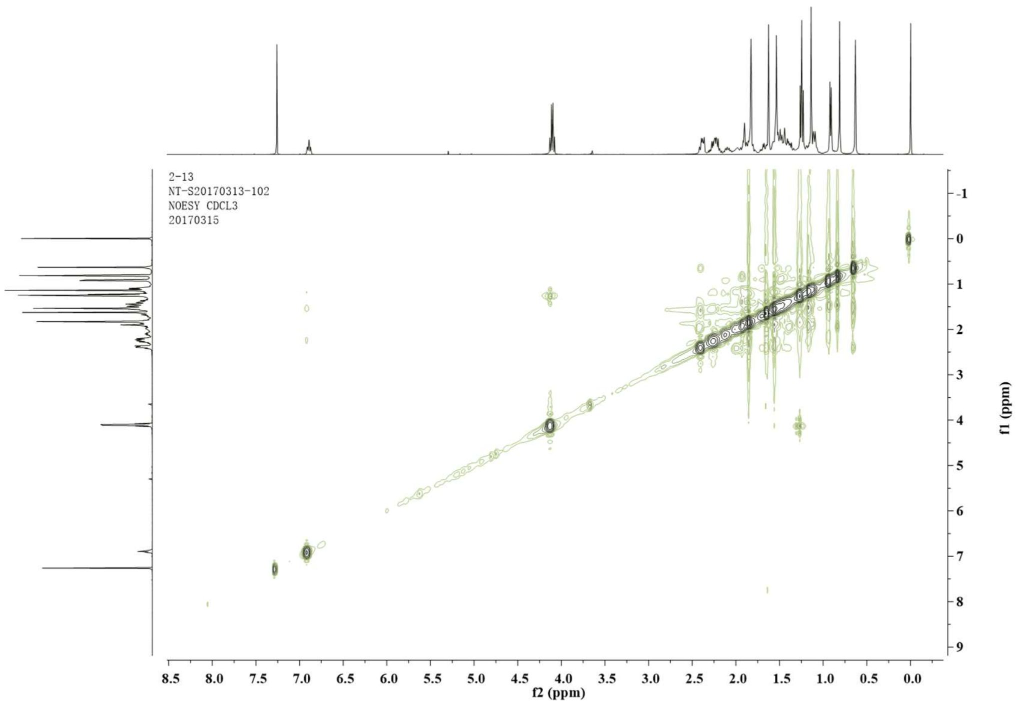

Figure S31. NOESY spectrum of compound **4**

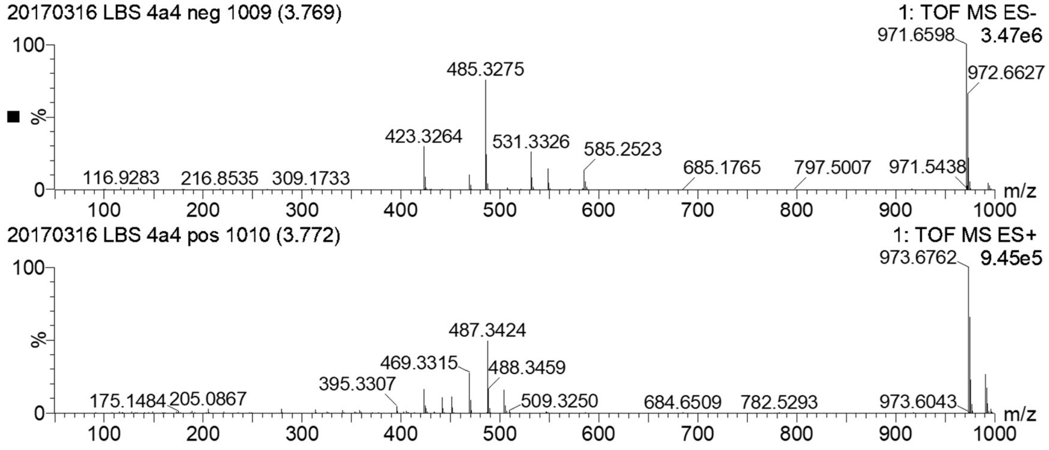

Figure S32. HR-ESI-MS spectrum of compound **5**

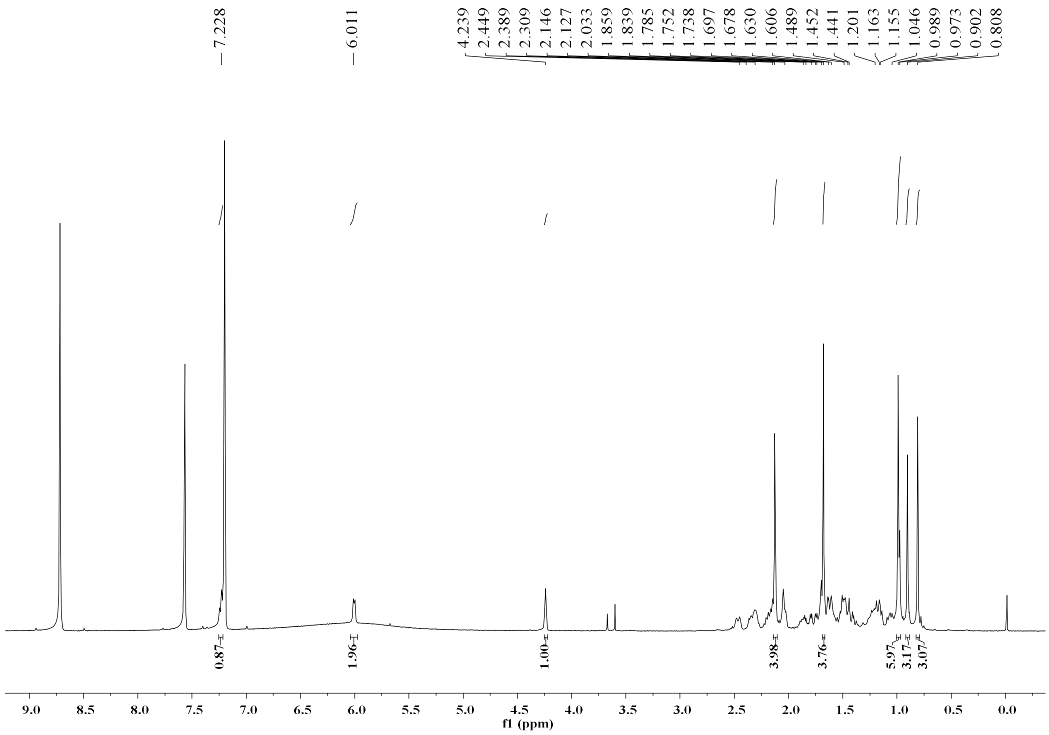

Figure S33. ^{1}H-NMR spectrum of compound **5**

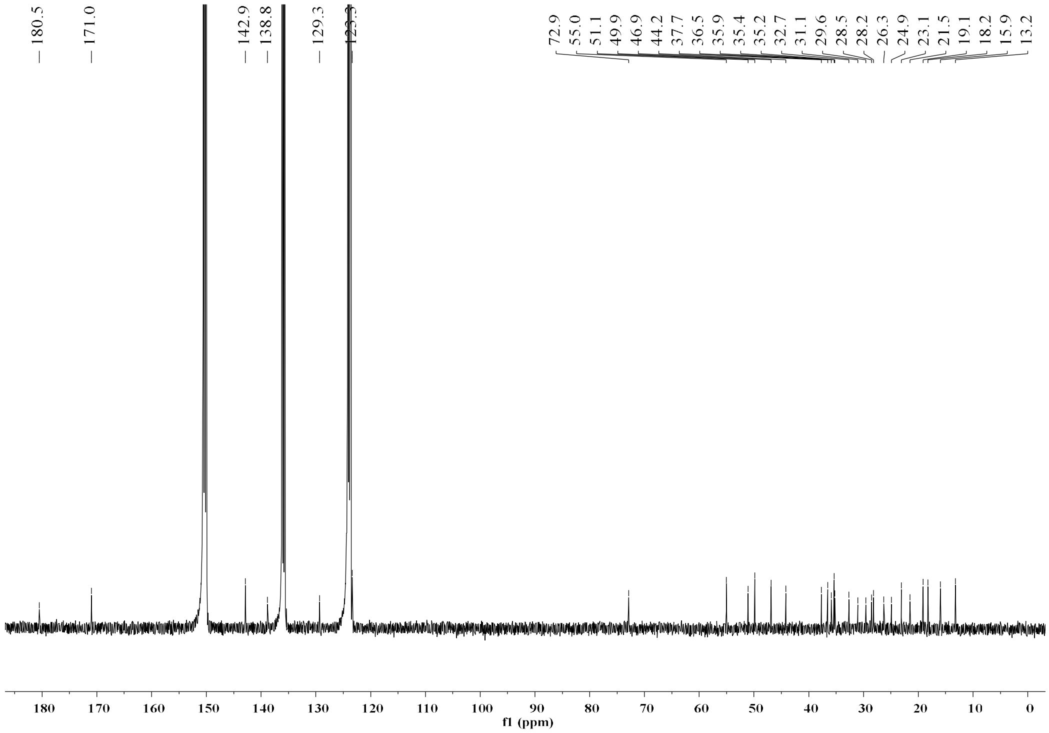

Figure S34. ^{13}C-NMR spectrum of compound **5**

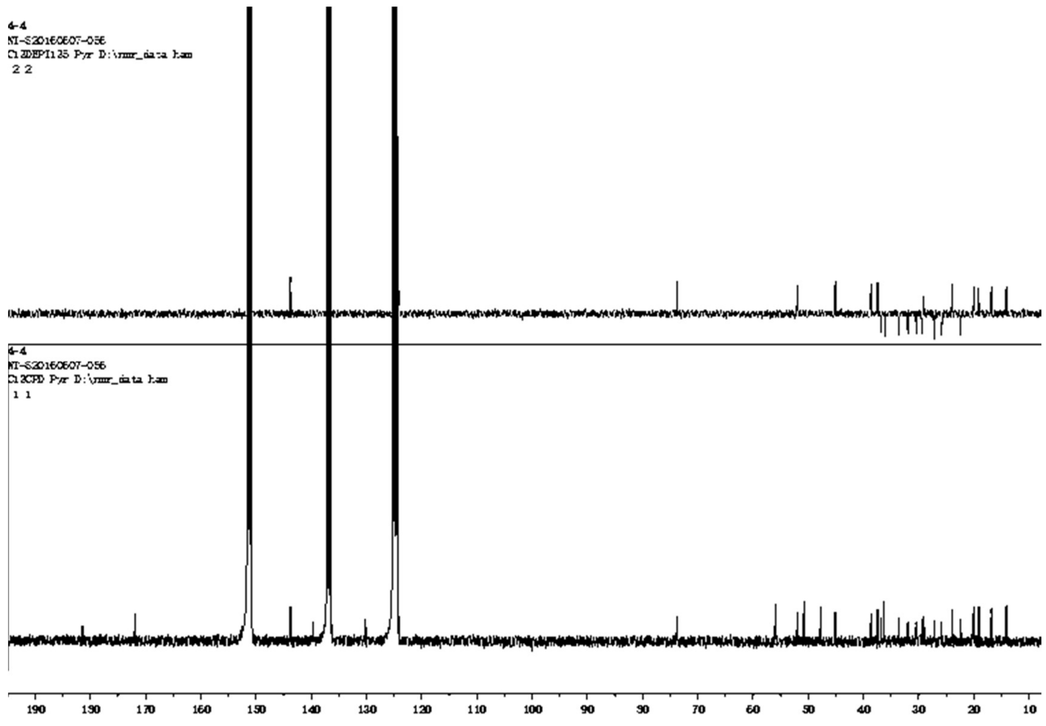

Figure S35. DEPT-135 spectrum of compound **5**

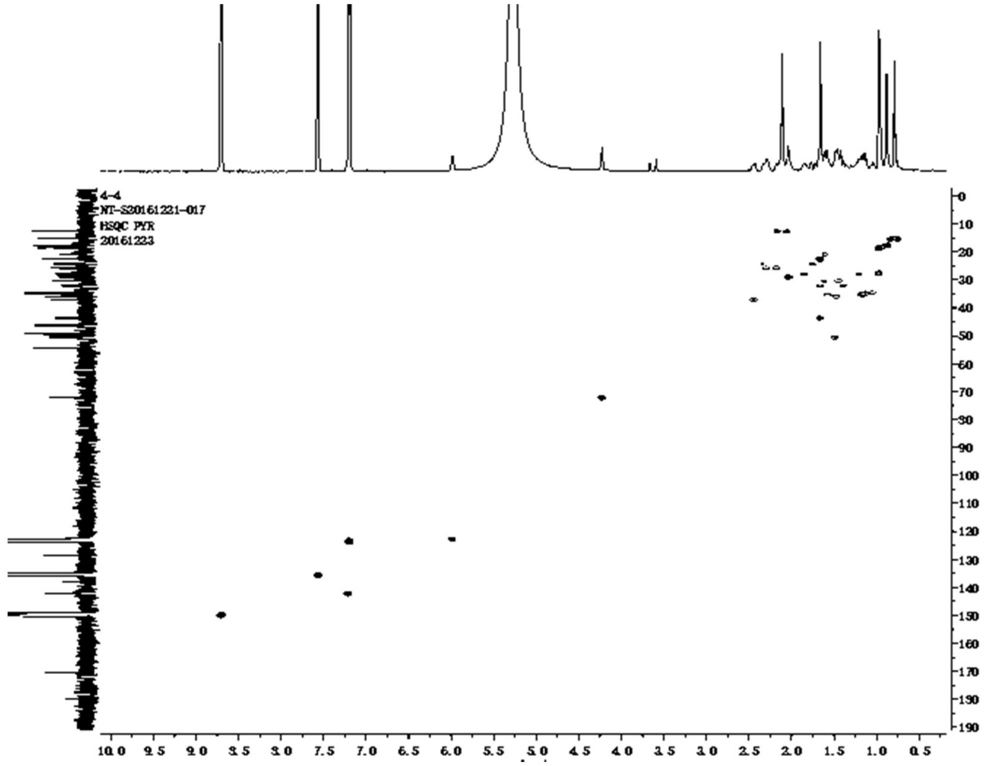

Figure S36. HSQC spectrum of compound **5**

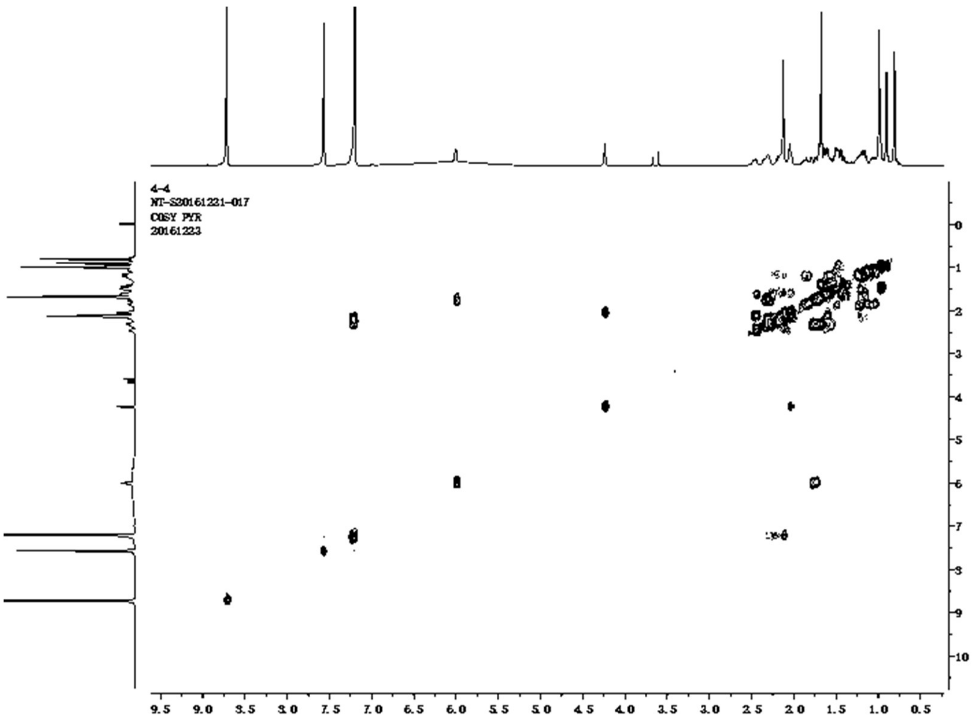

Figure S37. ^1H-^1H COSY spectrum of compound **5**

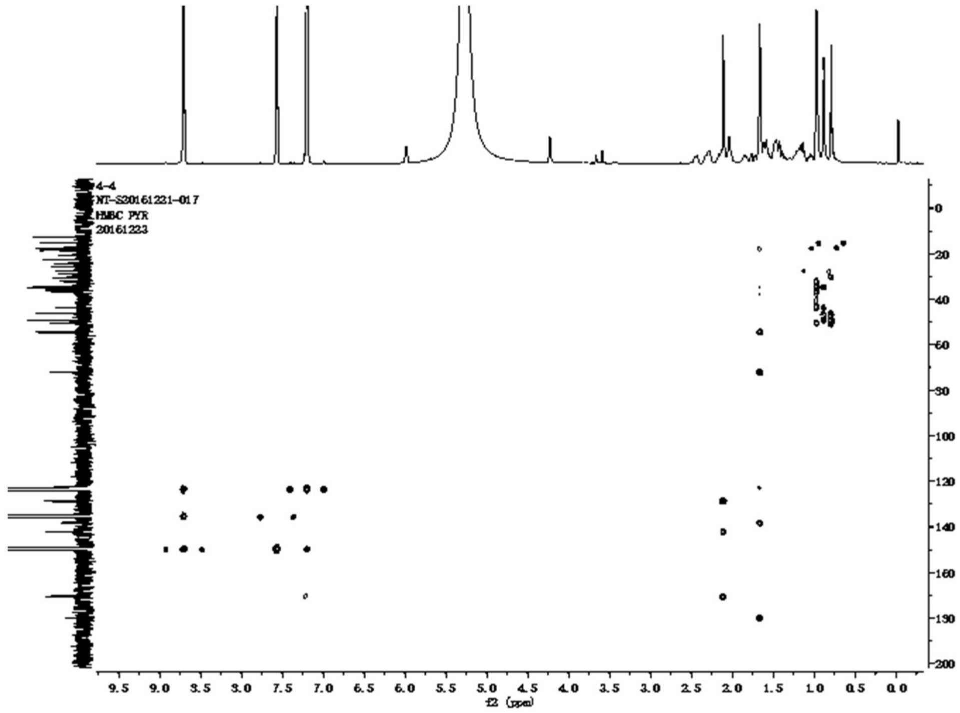

Figure S38. HMBC spectrum of compound **5**

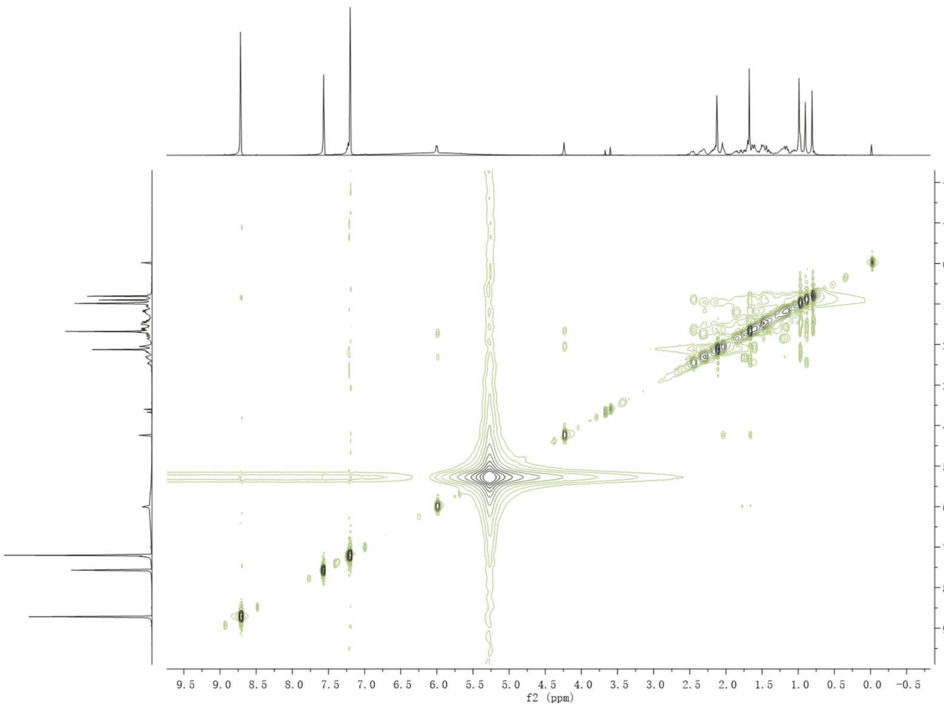

Figure S39. NOESY spectrum of compound **5**

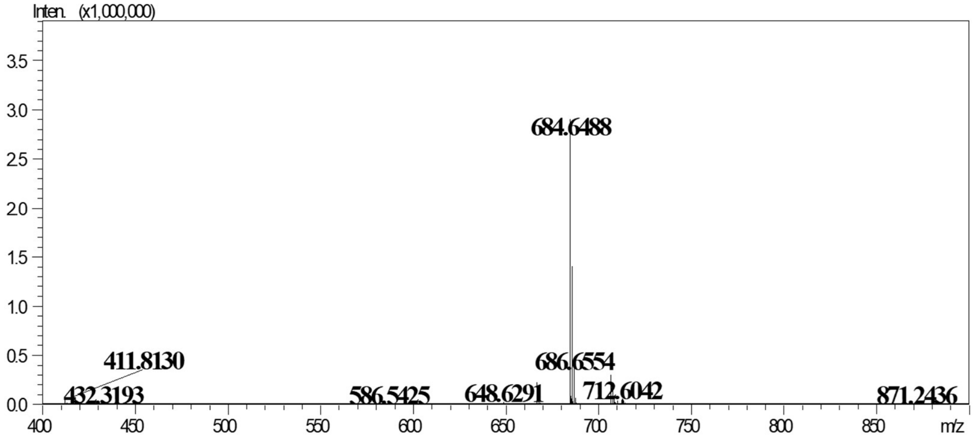

Figure S40. LC-IT-TOF-MS spectrum of compound **6** $[M+H]^+$

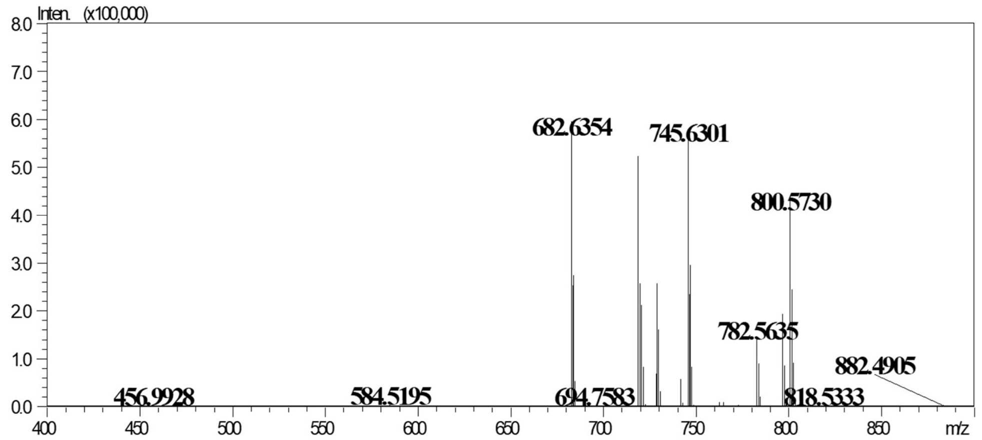

Figure S41. LC-IT-TOF-MS spectrum of compound **6** [M-H]$^-$

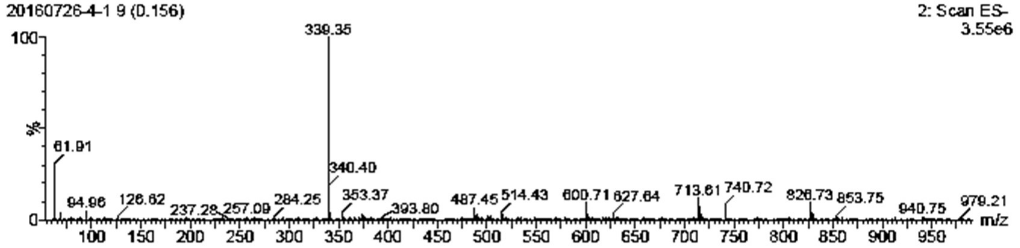

Figure S42. ESI-MS spectrum of compound **6**

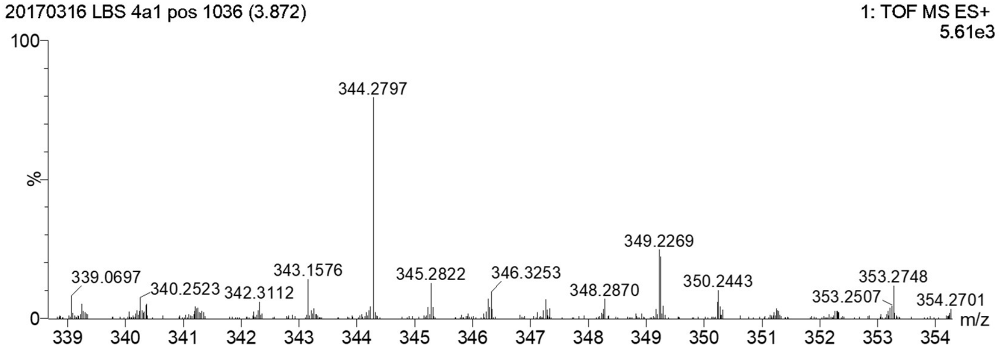

Figure S43. HR-ESI-MS spectrum of compound **6**

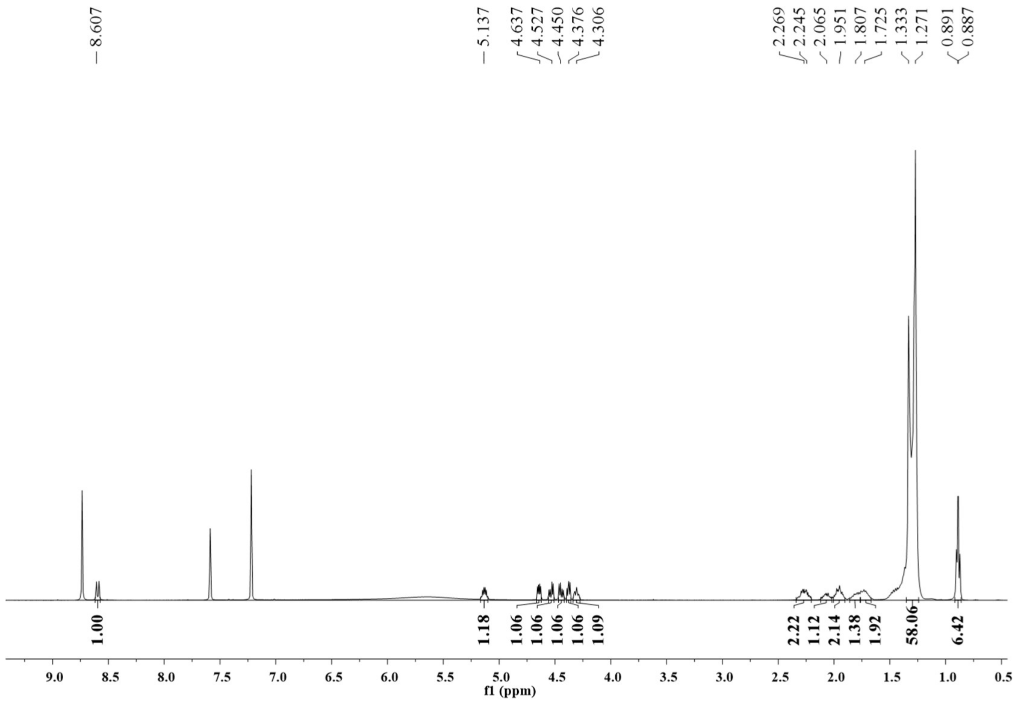

Figure S44. ^{1}H-NMR spectrum of compound **6**

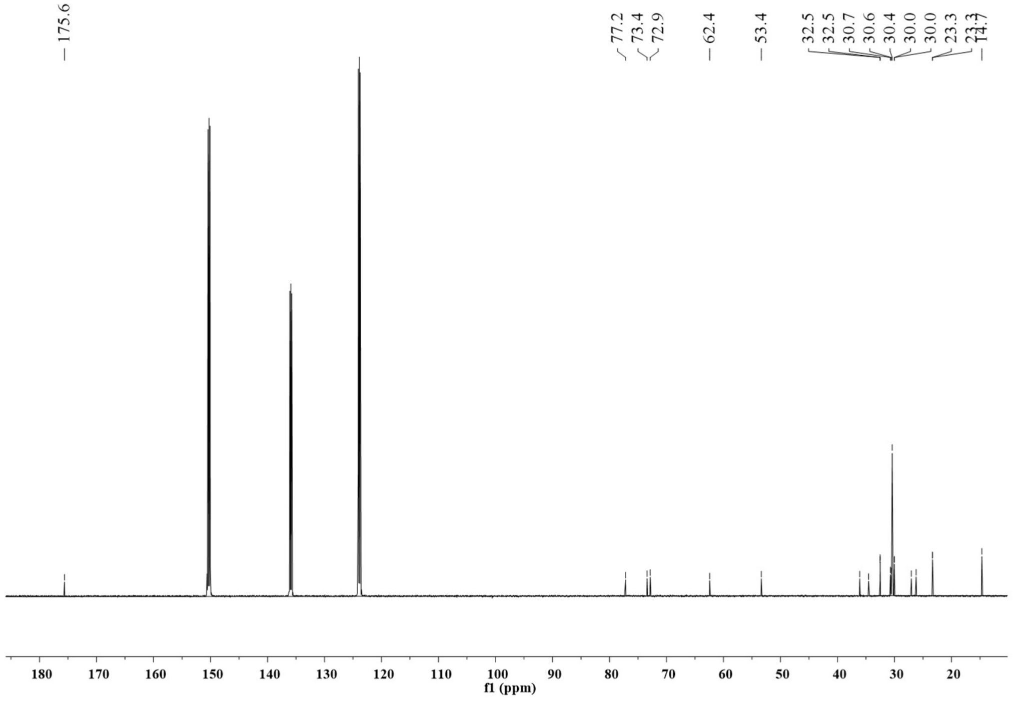

Figure S45. ^{13}C-NMR spectrum of compound **6**

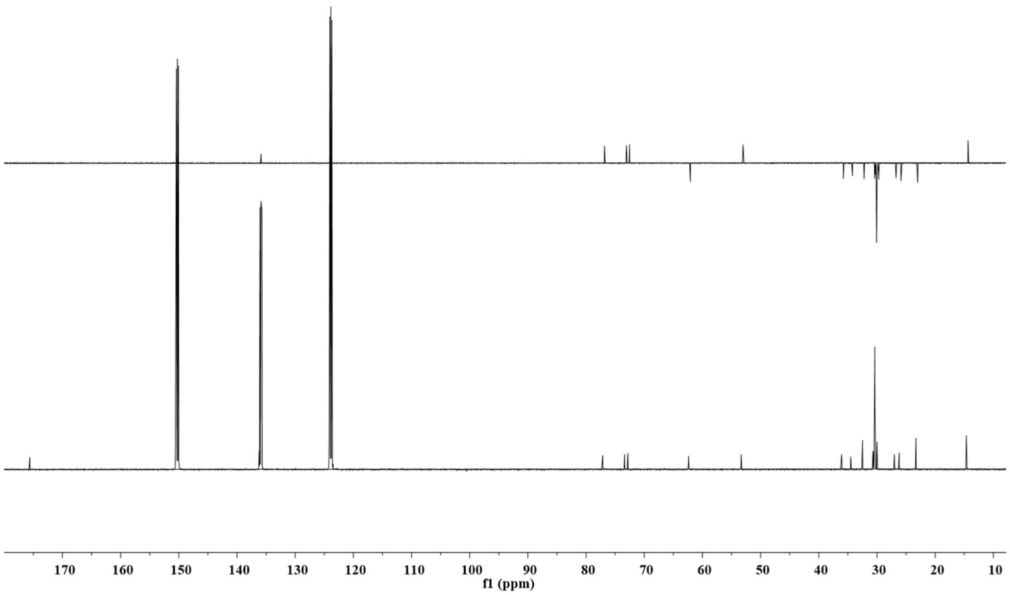

Figure S46. DEPT-135 spectrum of compound **6**

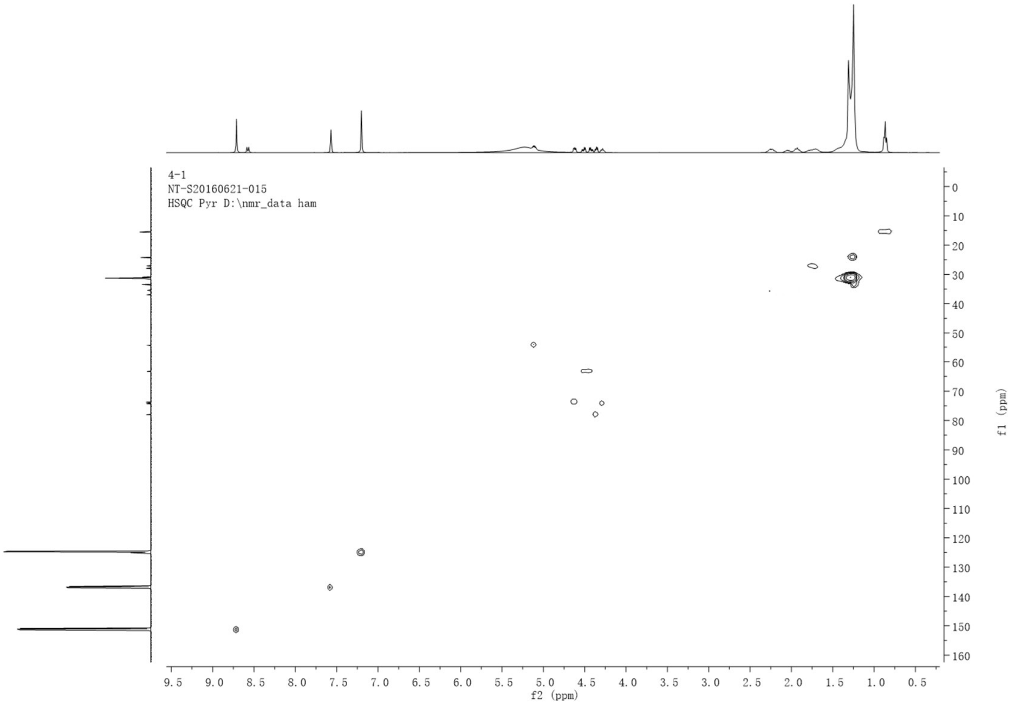

Figure S47. HSQC spectrum of compound **6**

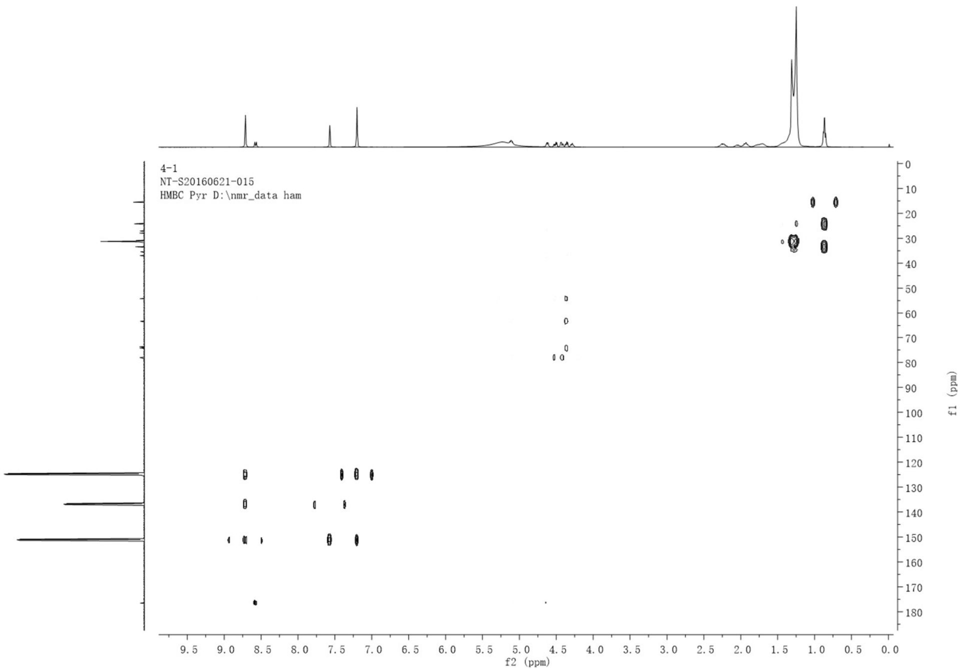

Figure S48. HMBC spectrum of compound **6**

Table 1. Identification of 15 isolates of 95% ethanol extract from the *R. vinosa* in positive ion mode.

No.	Compound	Calculated Value	Measured Value	Error (ppm)	Retention Time	Response Value
**1**	vinosane	251.1642	251.1642	0	3.34	34,512
**2**	rulepidadione C	251.1642	251.1634	−3	13.15	220,742
**3**	(24*E*)-3, 4-seco-cucurbita-4, 24-diene-26, 29-dioic acid-3-methyl ester	517.3524	517.3518	−1	25.91	22,767
**4**	(24*E*)-3, 4-seco-cucurbita-4, 24-diene-26-oic acid-3-ethyl ester	501.3938	501.3921	−3	31.26	104,871
**5**	(24*E*)-3β-hydroxycucurbita-5, 24-diene-26, 29-dioic acid	487.3418	487.3414	−1	22.5	232,364
**6**	(2*S*, 3*S*, 4*R*, 2′*R*)-2-(2′-hydroxydocosanoylamino) eicosane-1, 3, 4-triol	684.6501	684.6487	−2	32.05	25,299
**7**	(24*E*)-3, 4-secocucurbita-4, 24-diene-3, 26, 29-trioic acid	503.3367	503.3377	2	24.54	26,112
**8**	(24*E*)-3, 4-secocucurbita-4, 24-diene-3, 26-dioic acid	473.3625	473.3621	−1	28.4	32,493
**9**	(24*E*)-3β-hydroxycucurbita-5, 24-diene-26-oic acid	457.3676	457.3672	−1	28.92	97,362
**10**	rosacea acid B	457.3676	457.3665	−2	28.1	53,392
**11**	rosacea acid A	473.3625	473.3599	−5	32.13	91,186
**12**	7α, 8α, 13-trihydroxy-marasm-5-oic acid γ-lactone	267.1597	267.1591	−2	10.29	49,460
**13**	aristolone	219.1743	219.1739	−2	15.42	1,258,191
**14**	7, 8-dimethylalloxazine	243.0877	243.0872	−2	4.61	424,705
**15**	L-pyroglutamic acid	130.0499	130.0492	−5	0.69	25,781

Table 2. Identification of 15 isolates of 95% ethanol extract from the *R. vinosa* in negative ion mode.

No.	Compound	Calculated Value	Measured Value	Error (ppm)	Retention Time	Response Value
**1**	vinosane	249.1491	249.1491	0	3.35	11,148
**2**	rulepidadione C	249.1491	249.1491	0	13.15	35,665
**3**	(24*E*)-3, 4-seco-cucurbita-4, 24-diene-26, 29-dioic acid-3-methyl ester	515.3378	515.3376	−0	25.62	2,081,276
**4**	(24*E*)-3, 4-seco-cucurbita-4, 24-diene-26-oic acid-3-ethyl ester	499.3793	499.3797	1	31.27	2,701,378
**5**	(24*E*)-3β-hydroxycucurbita-5, 24-diene-26, 29-dioic acid	485.3272	485.3269	−1	22.51	237,837
**6**	(2*S*, 3*S*, 4*R*, 2′*R*)-2-(2′-hydroxydocosanoylamino) eicosane-1, 3, 4-triol	682.6355	682.6367	2	31.75	11,754
**7**	(24*E*)-3, 4-secocucurbita-4, 24-diene-3, 26, 29-trioic acid	501.3222	501.3223	0	24.29	175,827
**8**	(24*E*)-3, 4-secocucurbita-4, 24-diene-3, 26-dioic acid	471.3480	471.3481	0	28.4	4,757,652
**9**	(24*E*)-3β-hydroxycucurbita-5, 24-diene-26-oic acid	455.3536	455.3525	−2	29.07	2,466,945
**10**	rosacea acid B	455.3536	455.3531	−1	28.25	3,001,109
**11**	rosacea acid A	471.3480	471.3481	0	32.33	7508
**12**	7α, 8α, 13-trihydroxy-marasm-5-oic acid γ-lactone	265.1445	265.1443	−1	10.3	44,953
**13**	aristolone	217.1598	217.1590	−4	15.42	7760
**14**	7, 8-dimethylalloxazine	241.0731	241.0727	−1	4.61	72,213
**15**	L-pyroglutamic acid	128.0353	128.0349	−3	0.82	214,720
